# Developmental Changes in ANS Precision Across Grades 1–9: Different Patterns of Accuracy and Reaction Time

**DOI:** 10.3389/fpsyg.2021.589305

**Published:** 2021-03-24

**Authors:** Sergey Malykh, Yulia Kuzmina, Tatiana Tikhomirova

**Affiliations:** ^1^Department of Psychology, Lomonosov Moscow State University, Moscow, Russia; ^2^Psychological Institute of Russian Academy of Education, Moscow, Russia

**Keywords:** approximate number sense, non-symbolic comparison, speed-accuracy trade-off, general processing speed, numerical ratio effect

## Abstract

The main aim of this study was to analyze the patterns of changes in Approximate Number Sense (ANS) precision from grade 1 (mean age: 7.84 years) to grade 9 (mean age: 15.82 years) in a sample of Russian schoolchildren. To fulfill this aim, the data from a longitudinal study of two cohorts of children were used. The first cohort was assessed at grades 1–5 (elementary school education plus the first year of secondary education), and the second cohort was assessed at grades 5–9 (secondary school education). ANS precision was assessed by accuracy and reaction time (RT) in a non-symbolic comparison test (“blue-yellow dots” test). The patterns of change were estimated via mixed-effect growth models. The results revealed that in the first cohort, the average accuracy increased from grade 1 to grade 5 following a non-linear pattern and that the rate of growth slowed after grade 3 (7–9 years old). The non-linear pattern of changes in the second cohort indicated that accuracy started to increase from grade 7 to grade 9 (13–15 years old), while there were no changes from grade 5 to grade 7. However, the RT in the non-symbolic comparison test decreased evenly from grade 1 to grade 7 (7–13 years old), and the rate of processing non-symbolic information tended to stabilize from grade 7 to grade 9. Moreover, the changes in the rate of processing non-symbolic information were not explained by the changes in general processing speed. The results also demonstrated that accuracy and RT were positively correlated across all grades. These results indicate that accuracy and the rate of non-symbolic processing reflect two different processes, namely, the maturation and development of a non-symbolic representation system.

## Introduction

Humans and other species are equipped with the ability to perceive and process numerical information without counting and using symbols (e.g., Cantlon and Brannon, 2007; Agrillo et al., 2009; Nieder and Dehaene, 2009). Particularly, people can rapidly estimate and compare sets of objects based on their numerosities to determine the largest one or detect changes in numerosity. This ability can be supported by several systems of non-symbolic numerosity representations depending on the number of objects that should be perceived and the objects’ separation.

The first system is subitizing, which is the ability to precisely estimate numerosity in cases in which the number of objects is less than 4 (e.g., Revkin et al., 2008). Subitizing is based on an object tracking system and requires attentional and working memory resources (Olivers and Watson, 2008; Vetter et al., 2008; Burr et al., 2010). If the number of objects is larger than 3–4 and the boundaries of the objects are distinct, the Approximate Number System (ANS) is activated to estimate numerosity (Burr and Ross, 2008; Viswanathan and Nieder, 2013). Numerous studies have also demonstrated that when the number of objects increases and they have high density, objects are likely to be perceived as an inseparable texture, and the third system – texture-density discrimination – is activated (e.g., Anobile et al., 2016; Pomè et al., 2019).

Among the three systems of non-symbolic numerosity estimation, the ANS is more often discussed regarding its relations with symbolic math skills and developmental changes across the preschool and school years (e.g., Halberda et al., 2008, 2012; Halberda and Feigenson, 2008). Various studies have identified the following two main features of the ANS: its imprecision and its rapidity. ANS imprecision manifests as the proportion of errors (PE) and the existence of the Numerical Distance Effect (NDE) or Numerical Ratio Effect (NRE). The NRE or NDE indicate that the PE in a non-symbolic comparison test increases when the sets are closer to each other in numerosity (Sasanguie et al., 2011; Lyons et al., 2015). The size effect manifests as growing imprecision in non-symbolic comparison and estimation when the numerosity of sets of objects increases, while the ratio between the two sets remains the same (Dehaene, 2001).

### ANS Accuracy

To measure ANS, various tests are used. Among the most popular types of tests, in the numerosity comparison test, individuals compare two sets of objects (e.g., dots) and select the set that contains more objects (e.g., Gebuis and Reynvoet, 2012; Halberda et al., 2012; Norris and Castronovo, 2016). Different measures are used as indicators of ANS precision in non-symbolic comparison tests. In particular, accuracy (the proportion of correct answers) and the Weber fraction are the measures used in most studies (e.g., Halberda et al., 2012; Tosto et al., 2017). The Weber fraction reflects the smallest ratio between two sets of objects that can be reliably identified (Dietrich et al., 2015, 2016). In some cases, the NDE and NRE for accuracy can be calculated and are used as measures of ANS precision (e.g., Soltész et al., 2010; Lonnemann et al., 2011). Evidence suggests that accuracy-based measures are reliable and highly correlated with each other; thus, these measures can be used interchangeably (Inglis and Gilmore, 2014; Dietrich et al., 2016; Tosto et al., 2017). However, it has been shown that accuracy (proportion of correct answers) had the highest test-retest reliability among four possible measures of ANS precision (Inglis and Gilmore, 2014).

Usually, it is necessary to compare arrays of objects in a very short period. However, in different studies, the duration of the demonstration of the sets that must be compared varies. In particular, in the study by Halberda et al. (2008), the duration was 200 ms, whereas in the study by Smets et al. (2016), the duration was 1500 ms. Dietrich et al. (2016) manipulated the duration of the stimulus presentations (from 50 to 2400 ms) and demonstrated that ANS accuracy varied depending on the duration. It has been shown that the variance explained by the ratio between the two sets was higher under long reaction time (RT) conditions. As the authors noted, these results indicate that accuracy is more informative of the underlying numerosity representation under conditions with long presentation times.

### Speed of Non-symbolic Processing

To consider the speed of processing non-symbolic information, the mean (or median) RT (in all tasks or correct answers) is used. Particularly, it has been postulated that individuals who are able to estimate numerosity faster have a more precise ANS (e.g., Mussolin et al., 2010; Halberda et al., 2012; De Smedt et al., 2013). Several studies used other measures based on the RT. Particularly, in the study by Vanbinst et al. (2012), the NDE was calculated based on the RT. It was assumed that the RT-based NDE indicated the effect of distance on the children’s RT and that this effect was negative; hence, individuals who have higher ANS precision should demonstrate a lower NDE regarding RT.

However, RT-based measures are used less often than accuracy-based measures (Dietrich et al., 2016). Evidence suggests that RT-based measures (particularly the mean RT, NDE and NRE of RT) are not all correlated. In addition, accuracy-based measures are more informative regarding the underlying ANS acuity than RT-based measures (Dietrich et al., 2016). Particularly, it has been shown that there were no significant differences in RT between children with dyscalculia and children without such problems, whereas the differences in accuracy were significant (Piazza et al., 2010).

In addition to the low reliability of the measures based on RT, other methodological issues hinder the use of RT in ANS analyses. In particular, RT data usually violate the normal distribution assumption and demonstrate positive skewness. In addition, in some cases, in empirical data, influential values may distort the model fit (e.g., Baayen and Milin, 2010). As a normal distribution is an assumption of general linear models, some authors recommend applying different transformations to RT data to normalize the distribution (Whelan, 2008). However, other researchers do not recommend transforming RT data and demonstrate that transformation may not be beneficial or may distort the interpretation of the results (e.g., Ratcliff, 1993; Schramm and Rouder, 2019).

There are two different types of relationships between accuracy and RT and two different approaches to the interpretation of individual differences in RT (e.g., Dodonova and Dodonov, 2013). In the information-processing approach (Jensen, 2006), it is assumed that tasks are very simple and that errors are random. Hence, accuracy scores or PE do not significantly vary among individuals and cannot reflect individual differences in the ability to process non-symbolic information. In these cases, the RT is used to assess individuals’ ability. It has been postulated that the RT is negatively correlated with ability and that individuals with higher ability (e.g., a more precise ANS) can perform tasks faster. Considering this assumption, it is expected that accuracy and RT should be negatively correlated in a non-symbolic comparison test.

In the education testing approach, the tasks might vary in their difficulties; thus, accuracy or PE can reflect individuals’ ability. In such tests, the RT can also be used to measure individuals’ ability, but the relationship between accuracy and RT might be more complex than that in the information-processing approach. When the RT and accuracy reflect the same construct, it is expected that the RT and accuracy could be negatively correlated. However, in some cases, individuals may prefer accuracy over speed and demonstrate a speed-accuracy trade-off (Ratcliff et al., 2015). In this situation, the RT and accuracy are positively correlated, complicating the interpretation of the results of tests based on RT measures only.

The association between RT and accuracy in complex tests might vary depending on the task difficulty and individuals’ ability. Evidence suggests that in easy tasks, the RT and accuracy are negatively correlated, whereas in more difficult tasks, the RT and accuracy are positively correlated (e.g., Neubauer, 1990; Dodonova and Dodonov, 2013). Particularly, it was demonstrated that in the Raven test, there was a difference in RT, but not in accuracy, in response to easy items between high-ability and low-ability individuals. Concurrently, high-ability individuals differed from low-ability individuals in terms of the rate of change in accuracy in response to more difficult items, but no differences in RT changes were observed (Dodonova and Dodonov, 2013).

Regarding the ANS, evidence suggests that the RT and accuracy are positively correlated; accordingly, a speed-accuracy trade-off has been found (e.g., Dietrich et al., 2016). Dietrich et al. (2016) noted that if participants showed a speed-accuracy trade-off, the accuracy and RT provided controversial information regarding the ability to process numerosity information in a non-symbolic format. However, in some studies, a negative correlation was found between ANS accuracy and the RT (e.g., Soltész et al., 2010; Libertus et al., 2013). Hence, the relationships between accuracy and RT in a non-symbolic comparison test might change depending on the sample or test difficulty.

Although it has been shown that accuracy is more informative regarding ANS precision than RT, the RT can reflect an important aspect of non-symbolic representation. Particularly, the RT was found to explain 5–8% of the variance in math performance in addition to the variance explained by ANS accuracy (Libertus et al., 2011). Moreover, it has been shown that the speed of different tests of non-symbolic comparison formed a separate latent factor distinct from accuracy (Soltész et al., 2010). Some authors claimed that it is necessary to consider both accuracy and the RT in assessing the characteristics of cognitive processes (e.g., Ratcliff et al., 2015). In summary, previous findings revealed that accuracy and RT might reflect different processes and cannot be used interchangeably as measures of ANS precision (Dietrich et al., 2016). Hence, investigations of developmental changes in ANS precision require an estimation of changes in both accuracy and RT.

### Developmental Changes in ANS Accuracy and RT

Some evidence suggests that ANS precision increases throughout development. Most studies investigating developmental changes in the ANS have been performed based on changes in accuracy (e.g., Odic, 2018; Tikhomirova et al., 2019; Kuzmina et al., 2020) or Weber fraction (Halberda and Feigenson, 2008; Halberda et al., 2012). Weber fraction was found to decrease (e.g., Odic et al., 2013), whereas accuracy was found to increase across ages (e.g., Tikhomirova et al., 2019).

Although the hypothesis that ANS precision in adults is higher than that in children has been confirmed in various cross-sectional studies, longitudinal studies have cast doubt regarding the growth in ANS precision as a general phenomenon. Particularly, it has been demonstrated that growth in ANS precision slows by the end of elementary school (Tikhomirova et al., 2019). Latent growth models revealed that a significant proportion of pupils did not demonstrate growth in ANS accuracy (Tikhomirova et al., 2019). In addition, the increases in accuracy in non-symbolic comparison were found to be significant only among pupils with a high level of fluid intelligence or processing speed (PS) (Kuzmina et al., 2020).

Evidence suggests that the RT in non-symbolic comparison tests also changes across development. It has been demonstrated that adults have lower RTs in non-symbolic comparison tests than children (Halberda et al., 2012). In particular, Halberda and colleagues revealed that the RT rapidly decreased from the ages of 11 to 16 years, and then, the rate of change slowed, while accuracy continued to improve from the ages of 16 to 30 years (Halberda et al., 2012).

It has also been postulated that the development of non-symbolic representation precision is related to decreasing NDE or NRE (for a discussion, see Lyons et al., 2015). Particularly, adults demonstrated a lower distance effect than children (Halberda and Feigenson, 2008; Holloway and Ansari, 2008). Neurophysiological evidence further suggests that differences exist in the distance effect between adults and children. The amount of activation in the intraparietal sulcus (IPS) has been found to decrease as the numerical distance increases (Pinel et al., 2001). Ansari and Dhital (2006) demonstrated that adult participants exhibited greater effects of numerical distance in the left IPS than children. The authors suggested that these differences were related to developmental shifts from more controlled to more automatic processing of the numerical magnitude (Ansari and Dhital, 2006). It is possible that the development of ANS precision might involve changes in both accuracy and RT, reflecting improvement in general PS.

### Development of General PS

A large body of evidence suggests that general PS increases across development (e.g., Kail, 2000; Kail and Ferrer, 2007; Nettelbeck and Burns, 2010; Coyle et al., 2011). It has been demonstrated that exponential and quadratic models of changes in general PS fit the data better than other models (e.g., linear, hyperbolic, and inverse regression models). It has been hypothesized that the patterns of changes in PS (linear increase with non-linear decrease) are consistent with the patterns of quadratic changes in physical growth in childhood and adolescence (Kail and Ferrer, 2007). Improvement in general PS is associated with the process of myelination and white matter integrity across childhood (Mabbott et al., 2006; Scantlebury et al., 2014; Chevalier et al., 2015; Chopra et al., 2018).

Alternative theories regarding the developmental trends in PS and its relationships with the development of other cognitive functions have been developed. The global trend hypothesis posits that all cognitive, motor and perceptual processes develop at the same rate (e.g., Hale, 1990; Kail, 1991). Kail (2000) suggested that general mechanisms limit the speed of processing information regardless of the task specificity. Particularly, it has been shown that PS in tasks, such as mental addition, mental rotation and simple motor skills, improved across development at a common rate according to an exponential function (Kail, 1991).

The alternative local trend hypothesis posits that all components of information processing develop at different rates (Bisanz et al., 1979). It has also been hypothesized that the rate of change in the speed of cognitive processes might be domain-specific, whereas within one domain, all components develop at a common rate (Kail and Miller, 2006). For example, it has been shown that children aged between 9 and 14 years have a faster PS in language tasks than non-language tasks. However, the rate of change in the PS of non-language tasks was faster than that in language tasks (Kail and Miller, 2006).

Improvement in general PS affects further improvement in other cognitive functions, such as working memory, intelligence, inhibition, math skills and reasoning ability (e.g., Fry and Hale, 1996; Kail et al., 2016). In particular, the following development cascade has been demonstrated: the general PS affects further improvement in working memory and intelligence, which, in turn, might affect improvement in general PS (Fry and Hale, 1996; Nettelbeck and Burns, 2010). It has also been shown that improvement in general PS partially explains the changes in general intelligence and accuracy of non-symbolic representation (Pezzuti et al., 2019; Kuzmina et al., 2020). However, the extent to which the changes in non-symbolic PS are explained by the development of general PS is unknown. From the perspective of the global trend hypothesis, age-related changes in an individual’s speed in a non-symbolic comparison test should be explained by age-related changes in general PS. The local trend hypothesis implies that the patterns and rates of change in general and non-symbolic PS might differ.

### Current Study

Considering the complex relationships among accuracy, RT and ability level, we hypothesized that developmental changes in the ANS should be analyzed while considering developmental changes in both accuracy and RT. Moreover, in previous studies, a speed-accuracy trade-off was found in non-symbolic comparison tests, but the developmental changes in the relationship between accuracy and RT in non-symbolic comparison tests are unknown. As the NRE is a core feature of non-symbolic representation, it is crucial to estimate developmental changes by considering the NRE.

In summary, our research aims are as follows:

(1) To assess developmental changes in accuracy and RT in non-symbolic representation across the school years,
(2) To assess the extent to which developmental changes in accuracy and RT vary depending on the ratio between compared arrays,
(3) To estimate the developmental relationships between accuracy and RT in a non-symbolic comparison test, and
(4) To estimate the extent to which the changes in general PS may explain the changes in accuracy and RT in a non-symbolic comparison test.

## Materials and Methods

### Sample

This study was conducted using data collected for an ongoing longitudinal project named the “Cross-cultural Longitudinal Analysis of Student Success” (CLASS) project. For the aim of this study, two cohorts of schoolchildren studying in one school in the Moscow region were tested. This school was a state school with no selection of pupils.

The first cohort was tested from grade 1 to grade 5. In total, 313 pupils were tested, but some pupils participated less than three times due to illness and absence from school on the date of testing. As at least three time points are necessary to carefully estimate developmental trajectories and development relationships (e.g., Duncan and Duncan, 2009; Curran et al., 2010), the data of the schoolchildren who participated once or twice were removed from the analysis. The patterns of missing data in the sample were tested, and the missing completely at random (MCAR) assumption was confirmed by Little’s MCAR test (1988) (Little, 1988). This test was non-significant (chi-square distance = 69.49, df = 64, *p* = 0.30), indicating that the MCAR assumption held. Since the MCAR assumption held and the sample size was sufficient, list-wise deletion can be applied to obtain adequate parameter estimates (Coertjens et al., 2017). The remaining sample consisted of 260 pupils (49% were girls, the mean age in grade 1 was 7.84, range 6.81–8.86), 17% of the pupils participated three times, 44% of the pupils participated four times, and 39% of the pupils participated five times.

The second cohort was tested from grade 5 to grade 9. The initial sample consisted of 246 pupils. Meanwhile, some pupils participated in the survey less than three times. To assess the growth trajectories more precisely, we analyzed the data of the pupils who participated at least three times. The patterns of missing data in the sample were tested, and the MCAR assumption was confirmed by Little’s MCAR test (1988) (Little, 1988). This test was non-significant (chi-square distance = 57.77, df = 59, *p* = 0.52), indicating that the MCAR assumption held. The final sample consisted of 210 pupils (52% were girls, the mean age in grade 5 was 11.82 years, range 10.54–12.57), 11% of the pupils participated three times, 38% of the pupils participated four times, and 51% of the pupils participated five times.

This study received approval from the Ethics Committee of the Psychological Institute of the Russian Academy of Education. Parental informed and written consent was obtained prior to the data collection. Consent was obtained from the children orally.

### Procedures and Instruments

The pupils were assessed at the end of the academic year (April–May). All participants were tested in quiet settings within their school facilities by trained experimenters. All experimenters strictly used the same protocol and instructions for the test administration across all measurements. The pupils completed non-symbolic comparison and general PS tests in the computer form. The experiment was performed in a computer classroom in groups of 14–15 pupils. Each pupil sat in front of an individual monitor screen approximately 60 cm from the screen and performed the experiment independently. Each computer had a 17” LCD display with a resolution of 1440–900 pixels and a refresh rate of 60 Hz.

#### ANS

A non-symbolic comparison test was used to estimate ANS precision at each time point. The participants were presented arrays of yellow and blue dots in an intermixed format and varying in size and number. The task required the participants to judge whether the array contained more yellow or blue dots by pressing the corresponding keys on the keyboard (for the yellow dots, the participants pressed the “
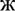
” key, corresponding to the “:” key on a QWERTY keyboard; for the blue dots, the participants pressed the “c” key, corresponding to the “c” key on a QWERTY keyboard). The following instructions were provided: “In this test, a number of circles will flash on the screen for less than half a second. The circles differ in size, and each circle is either yellow or blue. Your job is to judge whether you see more yellow or more blue circles flashing on the screen. If you think that there are more YELLOW circles, press “Y” on your keyboard. If you think that there are more BLUE circles, press “B” on your keyboard. Your decision must be based on the number of circles and not the sizes of the circles. In some trials, it may be difficult to judge. Don’t worry! Let your “number sense” guide you and go with your instinct. This test should take less than 10 min. You should try to complete the test in one session. However, if you prefer, you will be able to take a break at certain places in the test where you will see a “come back later” button. Remember, we are measuring speed and accuracy, so please respond as quickly as you can. Press the SPACE BAR to start.”

The stimuli included 150 static pictures, with the arrays of yellow and blue dots varying between 5 and 21 dots of each color and the ratios of the arrays of the two colors falling between 0.30 and 0.87. All trials can be divided into the following five ratio bins: 0.30–0.60 (23 trials), 0.61–0.75 (33 trials), 0.76–0.80 (29 trials), 0.81–0.84 (35 trials), and 0.85–0.87 (30 trials).

The presentation order was the same for all participants. Each stimulus flashed on the screen for 400 ms, and the maximum response time was 8 s. If no answer was given during this time, the answer was recorded as incorrect, and a message appeared on the screen to encourage the participant to press the space bar to continue to the next trial. The message disappeared after 20 s, and the next trial was displayed only after pressing the space bar. The task included a practice trial with two items and an option to repeat the practice trial.

In each trial, the cumulative area of the set containing more dots was larger than the cumulative area of the other set. The ratio of the cumulative areas of the two sets (the smallest area divided by the largest area) ranged between 0.30 and 0.87. In all trials, the average size of the yellow dots was equal to the average size of the blue dots.

To assess ANS precision, the following two measures were calculated: accuracy (proportion of correct answers) and RT (mean RT of the correct responses).

#### General PS

Processing speed was measured via modification of an RT test (Deary et al., 2001). In this version, the numbers 1, 2, 3, and 4 appeared 10 times each in a randomized order at random intervals between 1 and 3 s. The interval of 1 s was repeated 14 times, and intervals of 2 and 3 s between the presentations were repeated 13 times each. The task consisted of pressing the key corresponding to the number appearing on the screen as fast and accurately as possible. One series of numbers was used for all participants. The task started with instructions and a practice trial consisting of 6 items. The following instructions were provided: “This test should take only 2 or 3 min. You will need to complete the test in one go as there is no “come back later” option. We want to measure your speed, so please respond as quickly and as accurately as you can. You are going to see the numbers 1, 2, 3, and 4 flashing in the middle of the screen one at a time. Each time a number appears, press the matching key at the top of your keyboard as quickly as you can. To respond rapidly, you should position your left fingers on the keys “1” and “2” and your right fingers on the keys “3” and “4” as shown in the picture. Remember to only use the number keys at the top of the keyboard.” The practice trial could have been repeated. The time out for responses was 8 s. If no response was given during this time, the next trial followed. The mean RT of the correct responses was calculated as an indicator of PS. Lower RTs corresponded to higher general PS.

### Statistical Approach

First, we examined the accuracy and RT of the correct answers in each cohort and grade. To account for the non-symbolic comparison ratio dependence, we inspected the accuracy and RT in the following five ratio bins: 0.30–0.60 (23 trials), 0.61–0.75 (33 trials), 0.76–0.80 (29 trials), 0.81–0.84 (35 trials), and 0.85–0.87 (30 trials). The ratio was calculated as the smallest number divided by the largest number; thus, a larger ratio was associated with a decreasing distance between two numerosities that need to be compared. Next, the correlations between the accuracy and RT of the correct answers were estimated in each grade. To estimate the significance of the differences between the smallest and largest ratio bins in accuracy and RT, a paired-samples *t*-test was used.

To estimate the average and individual growth trajectories of non-symbolic representation, we used the mixed-effect growth approach (ME approach). The ME approach considers repeated measures that change over time “nested” in individuals. This approach allows researchers to estimate the average trajectory of the entire sample and individual-specific deviations from the average trajectory of each person. According to this framework, the intercept and the slope may vary across individuals, and this heterogeneity is described by the variance in the intercept and the slope.

We tested several models and used the likelihood ratio test (LR test) to choose the best-fitting model of the accuracy and mean RT of the correct answers as outcomes. For each cohort and outcome, several models were tested. The analysis started with testing the intercept-only model. This model estimates the intercept and between- and within-individual variance. The proportion of between-individual variance to the total variance (ICC) obtained from this model reflects the stability of outcomes across time. Higher ICC values correspond to greater between-individual variability and smaller within-individual variability (or greater time stability).

In several subsequent models, different patterns of changes were tested (linear changes and quadratic changes). We also tested random slope models and compared these models with a fixed slope model. A random slope model implies that the slope of the time variable varies across individuals. Hence, there were significant differences between individuals in the rate of change in ANS precision across the grades. In this model, the variance in the slope of the time variable and the covariance between the individual deviation of the slope and the intercept were estimated. To investigate the relationships between the changes in accuracy and RT, for each individual, the predicted growth in accuracy and RT were calculated, and the correlations between these measures were estimated.

Next, to estimate the extent to which the changes in general PS explain the changes in RT and accuracy in the non-symbolic comparison, the general PS was added to the model as a predictor of ANS, and RT and accuracy were the outcomes. If general PS explains the changes in ANS RT and accuracy, the coefficients of the “time” variable decrease or become insignificant. Finally, to compare the developmental patterns of general and non-symbolic PS, we estimated and compared the growth trajectories of general PS and non-symbolic PS.

## Results

### Descriptive Statistics

The descriptive statistics of the accuracy and RT in the non-symbolic comparison test in the whole test and five ratio bins are presented in Table 1. The results revealed that across all grades, the highest accuracy was obtained with the smallest proportion (for ratio 0.30–0.60).

**TABLE 1:** Descriptive statistics for accuracy and RT for all trials and for five ratio bins.

Grade	Accuracy (proportion of correct answers)											
	All		Bin1 0.30–0.60		Bin2 0.61–0.75		Bin3 0.76–0.80		Bin4 0.81–0.84		Bin5 0.85–0.87	
	M	SD	M	SD	M	SD	M	SD	M	SD	M	SD
**Cohort 1**												
1	0.63	0.09	0.75	0.17	0.66	0.12	0.60	0.11	0.58	0.11	0.58	0.10
2	0.65	0.08	0.79	0.15	0.68	0.12	0.63	0.12	0.60	0.10	0.61	0.10
3	0.68	0.08	0.82	0.12	0.71	0.11	0.65	0.11	0.62	0.10	0.62	0.10
4	0.69	0.08	0.85	0.13	0.73	0.12	0.67	0.11	0.63	0.10	0.62	0.11
5	0.69	0.08	0.85	0.12	0.72	0.11	0.67	0.10	0.63	0.09	0.63	0.11
**Cohort 2**												
5	0.67	0.09	0.83	0.14	0.70	0.12	0.65	0.12	0.62	0.11	0.61	0.11
6	0.69	0.09	0.84	0.15	0.72	0.13	0.66	0.12	0.63	0.09	0.64	0.10
7	0.69	0.09	0.84	0.14	0.73	0.13	0.66	0.11	0.65	0.10	0.63	0.10
8	0.73	0.08	0.89	0.12	0.76	0.12	0.70	0.10	0.66	0.10	0.67	0.10
9	0.75	0.07	0.92	0.09	0.80	0.11	0.72	0.09	0.68	0.09	0.67	0.10

**Grade**	**RT for correct answers (sec.)**											
	**All**		**Bin1 0.30–0.60**		**Bin2 0.61–0.75**		**Bin3 0.76–0.80**		**Bin4 0.81–0.84**		**Bin5 0.85–0.87**	
	**M**	**SD**	**M**	**SD**	**M**	**SD**	**M**	**SD**	**M**	**SD**	**M**	**SD**

**Cohort 1**												
1	1.51	0.51	1.51	0.46	1.49	0.53	1.51	0.56	1.54	0.58	1.51	0.59
2	1.37	0.39	1.38	0.39	1.35	0.44	1.37	0.43	1.37	0.44	1.40	0.46
3	1.23	0.30	1.20	0.28	1.22	0.35	1.23	0.33	1.25	0.36	1.25	0.36
4	1.10	0.27	1.07	0.25	1.10	0.30	1.09	0.30	1.13	0.31	1.13	0.32
5	1.01	0.26	0.96	0.21	1.01	0.28	1.01	0.28	1.02	0.29	1.03	0.28
**Cohort 2**												
5	1.04	0.25	1.03	0.26	1.04	0.28	1.02	0.28	1.06	0.30	1.06	0.30
6	0.93	0.23	0.90	0.22	0.92	0.25	0.94	0.25	0.95	0.27	0.95	0.26
7	0.85	0.22	0.82	0.19	0.85	0.26	0.85	0.23	0.88	0.26	0.87	0.27
8	0.88	0.19	0.81	0.16	0.90	0.23	0.92	0.24	0.94	0.25	0.92	0.24
9	0.89	0.17	0.81	0.14	0.89	0.20	0.90	0.20	0.93	0.21	0.93	0.21

Significant positive correlations were observed between the RT of the correct answers and accuracy in grades 1–5 in Cohort 1 and grades 5–9 in Cohort 2 (Table 2). Hence, a speed-accuracy trade-off was found in each grade, although the values of the correlations varied across the grades.

**TABLE 2:** Correlations between accuracy and mean RT for correct answers.

Grade	Correlation between accuracy and RT for correct answers
**Cohort 1**	
Grade 1	0.53***
Grade 2	0.45***
Grade 3	0.32***
Grade 4	0.52***
Grade 5	0.42***
**Cohort 2**	
Grade 5	0.50***
Grade 6	0.47***
Grade 7	0.68***
Grade 8	0.55***
Grade 9	0.39***

***p < 0.001.

The descriptive statistics of general PS are presented in Supplementary Material Table 3.

### Estimation of the Ratio Dependence in the ANS Accuracy and RT

A paired-samples *t*-test was conducted to estimate the significance of the differences in accuracy between the smallest ratio bin (0.30–0.60) and the largest ratio bin (0.85–0.87). The analysis revealed that the difference in accuracy between the smallest and largest ratio bins was significant in both cohorts across all time points (Table 3). The analysis also demonstrated that in both cohorts, the effect size of the difference between the two ratio bins increased across time.

**TABLE 3:** Results of paired-sample *t*-test for differences in ANS accuracy and RT between the smallest and the largest ratio bins.

Grade	Bin1 (ratio 0.30–0.60)		Bin5 (ratio 0.85–0.87)		Mean difference (95% CI)	t	df	Effect size (Cohen’s *d*)
	M	SD	M	SD				
**Accuracy (Cohort 1)**								
1	0.75	0.17	0.58	0.10	0.17 (0.15; 0.19)	15.61***	179	1.23
2	0.79	0.15	0.61	0.10	0.18 (0.16; 0.20)	18.24***	218	1.43
3	0.82	0.12	0.62	0.10	0.21 (0.19; 0.22)	25.99***	227	1.87
4	0.85	0.13	0.62	0.11	0.22 (0.21; 0.23)	26.61***	238	1.86
5	0.85	0.12	0.63	0.11	0.22 (0.21; 0.24)	29.72***	232	1.95
**Accuracy (Cohort 2)**								
5	0.83	0.14	0.61	0.11	0.21 (0.19; 0.23)	24.16***	186	1.68
6	0.84	0.15	0.64	0.10	0.20 (0.18; 0.20)	18.00***	166	1.60
7	0.84	0.14	0.63	0.10	0.20 (0.18; 0.22)	20.48***	182	1.63
8	0.89	0.12	0.67	0.10	0.22 (0.21; 0.24)	27.90***	198	2.05
9	0.92	0.09	0.67	0.10	0.25 (0.23; 0.26)	29.59***	189	2.52
**RT (sec.) (Cohort 1)**								
1	1.51	0.46	1.51	0.59	−0.00 (−0.05; 0.05)	−0.03	179	−0.002
2	1.38	0.39	1.40	0.46	−0.01 (−0.05; 0.03)	−0.47	218	−0.02
3	1.20	0.28	1.25	0.36	−0.05 (−0.08; −0.02)	−3.02**	227	−0.16
4	1.07	0.25	1.13	0.32	−0.06 (−0.09; −0.03)	−4.60***	238	−0.21
5	0.96	0.21	1.03	0.28	−0.07 (−0.09; −0.04)	−5.36***	232	−0.27
**RT (sec.) (Cohort 2)**								
5	1.03	0.26	1.06	0.30	−0.02 (−0.06; 0.01)	−1.27	186	−0.08
6	0.90	0.22	0.95	0.26	−0.05 (−0.08; −0.02)	−3.16**	166	−0.19
7	0.82	0.19	0.87	0.27	−0.04 (−0.07; −0.02)	−3.28**	182	−0.19
8	0.81	0.16	0.92	0.24	−0.08 (−0.10; −0.06)	−7.40***	198	−0.41
9	0.81	0.14	0.93	0.21	−0.12 (−0.14; −0.09)	−10.56***	189	−0.65

***p < 0.001, **p < 0.01.

The analysis of the difference in the ANS RT between the ratio bins revealed that the difference was insignificant in grades 1–2 in Cohort 1 and grade 5 in Cohort 2 (Table 3). These results revealed that ANS precision varied depending on the ratio between the two compared arrays, although these differences mostly manifested in accuracy rather than RT.

### Developmental Changes in ANS Accuracy

The growth trajectories of ANS accuracy measured by the non-symbolic comparison test were estimated in Cohort 1 (grades 1–5) and Cohort 2 (grades 5–9) separately. The results of Cohort 1 are presented in Table 4.

**TABLE 4:** Cohort 1: results of ME growth models for changes in ANS accuracy from grade 1 to grade 5.

Variables	Baseline	Model 1	Model 2	Model 3
	
	Intercept-only	Linear growth	Non-linear growth	Model with random slope
	
	B (s.e.)	B (s.e.)	B (s.e.)	B (s.e.)
**Fixed effect**				
Constant	0.67*** (0.004)	0.63*** (0.005)	0.62*** (0.006)	0.62*** (0.006)
Time		0.02*** (0.001)	0.036*** (0.005)	0.037*** (0.004)
Time^2^			−0.005*** (0.001)	−0.005*** (0.001)
**Random effect**				
Intercept variance	0.003	0.003	0.003	0.004
Residuals	0.005	0.004	0.004	0.004
Slope variance (time)				0.0002
Covariance between intercept and slope				−0.0004
Log-likelihood	11191.9	1255.53	1262.74	1270.79
LR test (Δ df)		127.27*** (1)	14.42*** (1)	16.11*** (2)
ICC	0.36			

***p < 0.001.

The results of the ME growth model of Cohort 1 revealed that the model with non-linear changes and a random slope fit the data better than the models with linear changes or a fixed slope. The values of the coefficients of the variables “time” and “time^2^” demonstrated that ANS accuracy increased from grade 1 to grade 5, but growth slowed after grade 3. The results of post-estimation revealed that there was no difference in average predicted accuracy between grades 3 – 5 (Supplementary Table 1). The covariance between the intercept and slope at the individual level was significant and negative, indicating that the pupils who had a higher level of accuracy at grade 1 demonstrated less growth (Supplementary Figure 1).

The results of the changes in accuracy in Cohort 2 (grades 5–9) are presented in Table 5. The results of the pupils in grades 5–9 revealed that the model with non-linear changes and a random slope fit the data better than the model with linear changes and a fixed slope (Supplementary Figure 2). The results of post-estimation indicated that predicted average accuracy did not increase from grade 5 to grade 7 but increased later (grades 8–9) (Supplementary Table 2).

**TABLE 5:** Cohort 2: results of ME growth model for changes in ANS accuracy from grade 5 to grade 9.

Variables	Baseline	Model 1	Model 2	Model 3
	
	Intercept-only	Linear growth	Non-linear growth	Model with random slope
	
	B (s.e.)	B (s.e.)	B (s.e.)	B (s.e.)
**Fixed effect**				
Constant	0.71*** (0.004)	0.67*** (0.005)	0.68*** (0.006)	0.68*** (0.006)
Time		0.018*** (0.001)	0.005 (0.005)	0.005 (0.006)
Time^2^			0.003** (0.001)	0.003** (0.001)
**Random effect**				
Intercept variance	0.005	0.004	0.004	0.005
Residuals	0.003	0.003	0.003	0.003
Slope variance (time)				0.0002
Covariance between intercept and slope				−0.0006
Log-likelihood	1005.68	1075.70	1079.42	1088.91
LR test (Δ df)		140.03*** (1)	7.44** (1)	20.31*** (2)
ICC	0.39			

***p < 0.001, ** *p* < 0.01.

**FIGURE 1:**
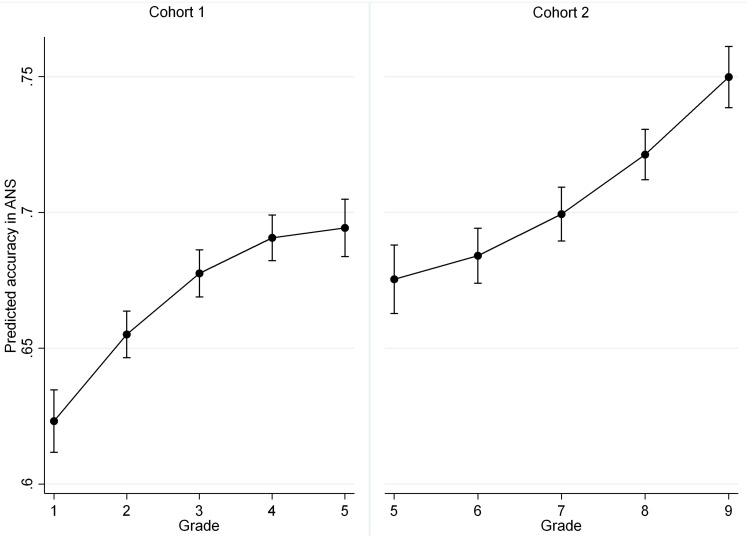
Average predicted trajectories of changes in ANS accuracy for two cohorts (with 95% CI).

The comparison of the average accuracy in grade 5 in both cohorts revealed that there is no difference in accuracy in grade 5 between the two cohorts. The analysis of the average growth trajectories in grades 1–5 and 5–9 indicated that accuracy was relatively stable from grade 3 to grade 7 (Figure 1).

### Changes in the Speed of Non-symbolic Processing

Furthermore, we estimated the patterns of the changes in the speed of non-symbolic processing as measured by the RT in the non-symbolic comparison task. The results of the ME growth model of the RT of the correct answers in Cohort 1 are demonstrated in Table 6.

**TABLE 6:** Cohort 1: results of ME growth models for changes in ANS RT (in sec.) from grade 1 to grade 5.

Variables	Baseline	Model 1	Model 2	Model 3
	
	Intercept-only	Linear growth	Non-linear growth	Model with random slope
	
	B (s.e.)	B (s.e.)	B (s.e.)	B (s.e.)
**Fixed effect**				
Constant	1.22*** (0.02)	1.49*** (0.02)	1.52*** (0.02)	1.49*** (0.03)
Time		−0.13*** (0.01)	−0.17*** (0.02)	−0.13*** (0.01)
Time^2^			0.01 (0.01)	
**Random effect**				
Intercept variance	0.04	0.05	0.05	0.15
Residuals	0.11	0.08	0.08	0.06
Slope variance (time)				0.01
Covariance between intercept and slope (time)				−0.03
Log-likelihood	−469.16	−300.47	−298.65	−245.37
LR test (Δ df)		337.38*** (1)	3.65 (1)	112.46*** (2)
ICC	0.26			

***p < 0.001.

The analysis revealed that the RT of the correct answers decreased from grade 1 to grade 5 according to a linear pattern as the model with non-linear changes did not fit the data better than the model with linear changes. The model with a random slope fit the data better than the model with a fixed slope. The covariance between the individual intercept and slope was negative, indicating that individuals with a larger RT in grade 1 had greater changes in RT (Supplementary Figure 3).

The results of the ME growth models of the RT in the non-symbolic comparison test in Cohort 2 (grade 5–grade 9) are presented in Table 7.

**TABLE 7:** Cohort 2: results of ME growth models for changes in ANS RT (in sec.) from grade 5 to grade 9.

Variables	Baseline	Model 1	Model 2	Model 3	Model 3a
	
	Intercept-only	Linear growth	Non-linear growth	Model with random slope1	Model with random slope2
	
	B (s.e.)	B (s.e.)	B (s.e.)	B (s.e.)	B (s.e.)
**Fixed effect**					
Constant	0.92*** (0.01)	1.00*** (0.01)	1.05*** (0.01)	1.05*** (0.02)	1.04*** (0.02)
Time		−0.04*** (0.004)	−0.14*** (0.01)	−0.14*** (0.02)	−0.14*** (0.02)
Time^2^			0.02*** (0.003)	0.03*** (0.003)	0.02*** (0.003)
**Random effect**					
Intercept variance	0.02	0.02	0.02	0.04	0.04
Residuals	0.03	0.03	0.03	0.02	0.02
Slope variance (time)				0.002	0.02
Slope variance (time^2^)					0.001
Covariance between intercept and slope (time)				−0.007	−0.02
Covariance between intercept and slope (time^2^)					0.002
Covariance between slope (time) and slope (time^2^)					−0.004
Log-likelihood	131.94	172.40	198.99	219.75	234.05
LR test (Δ df)		80.92*** (1)	53.19*** (1)	41.51*** (2)	28.59*** (3)
ICC	0.31				

***p < 0.001.

The analysis results revealed that the model with non-linear changes and a random slope for the variables “time” and “time^2^” fit the data better than the other models. The RT decreased from grade 5 to grade 7 and then decreased more slowly thereafter. The post-estimation results revealed that there was no difference in RT between grades 7–9 (Supplementary Table 2). The covariance between the individual intercept and the slope of the variable “time” was negative, while the covariance between the intercept and slope of “time^2^” was positive. This finding indicated that the individuals who had a larger RT in grade 5 demonstrated a larger decrease in RT from grade 5 to grade 6 and a larger deceleration later (Supplementary Figure 4).

**FIGURE 2:**
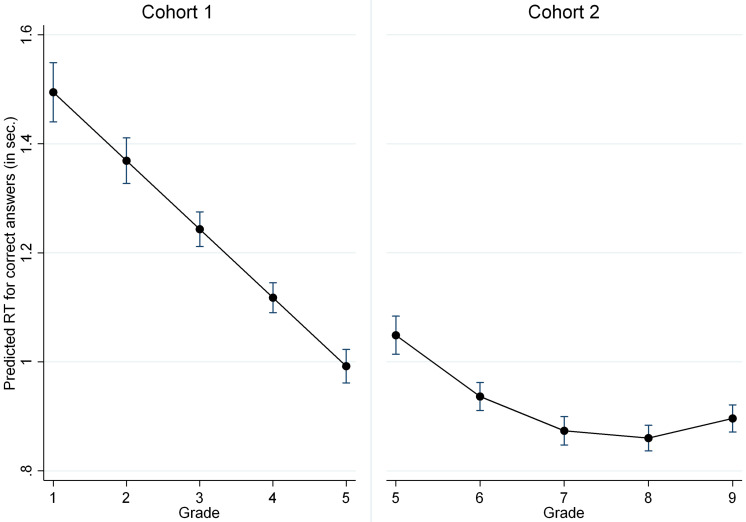
Average predicted trajectories of changes in ANS RT for two cohorts (with 95% CI).

The comparison of the average predicted trajectories of the RT in the non-symbolic comparison test and post-estimation revealed that the changes in RT across grades 5–9 were less prominent than those across grades 1–5 (Figure 2).

Notably, no significant differences in RT in the non-symbolic comparison test in grade 5 were observed between the two cohorts.

### Changes in Accuracy and RT in Small and Large Ratio Bins

Considering the difference in accuracy between the small and large ratio bins, we inspected the growth trajectories in the easiest ratio (0.30–0.60) and hardest ratio (0.85–0.87). The results of Cohort 1 are presented in Table 8. The results revealed that in both ratio bins, accuracy increased according to a non-linear pattern. In the easiest ratio, the model with a random slope fit the data better than the model with a fixed slope. Hence, there was significant between-individual variability in the rate of change in accuracy. In the large ratio bin, the model with a random slope did not fit the data better than the model with a fixed slope. Therefore, the individual differences in the rate of change were not significant.

**TABLE 8:** Cohort 1: results of ME growth models for changes in ANS accuracy for the easiest (0.30–0.60) and hardest (0.85–0.87) ratio bins from grade 1 to grade 5.

Variables	Bin 1: 0.30–0.60		Bin 5: 0.85–0.87	
	Baseline	Model 3	Baseline	Model 2
	
	Intercept-only	Non-linear growth with random slope	Intercept-only	Non-linear growth with fixed slope
	
	B (s.e.)	B (s.e.)	B (s.e.)	B (s.e.)
**Fixed effect**				
Constant	0.82*** (0.01)	0.74*** (0.01)	0.61*** (0.004)	0.58*** (0.01)
Time		0.05*** (0.01)		0.03*** (0.007)
Time^2^		−0.007** (0.002)		−0.004* (0.002)
**Random effect**				
Intercept variance	0.0053	0.012	0.0015	0.0016
Residuals	0.0147	0.011	0.009	0.009
Slope variance (time)		0.00058		
Covariance between intercept and slope		−0.00197		
Log-likelihood	637.07	700.82	921.67	937.94
LR test (Δ df)		19.11*** (2) vs. model with fixed slope		3.86* (1) vs. model with linear growth
ICC	0.27		0.14	

***p < 0.001, **p < 0.01, *p < 0.05.

Although the pattern of the changes was the same in the two ratio bins, the coefficient of the variable “time” was higher in the easiest ratio, indicating a larger growth in accuracy in the easiest ratio. However, the absolute value of the negative regression coefficient of the “time squared” variable was smaller in the hardest ratio, indicating less slowing in growth (Supplementary Figure 5).

The results of the analysis of the changes in accuracy in Cohort 2 (grades 5–9) in two ratio bins separately are presented in Table 9. The analysis revealed that in the easiest ratio, accuracy increased according to a non-linear pattern. In particular, post-estimation revealed that accuracy did not increase from grade 5 to grade 7, but the difference between grade 7 and 8 became significant. The analysis also revealed that in the hardest ratio, accuracy increased according to a linear pattern. Hence, in the second cohort, the patterns of accuracy changes differed between the easiest and hardest ratio bins (Supplementary Figure 6).

**TABLE 9:** Cohort 2: results of ME growth models for changes in ANS accuracy for the easiest (0.30–0.60) and hardest (0.85–0.87) ratio bins from grade 5 to grade 9.

Variables	Bin 1: 0.30–0.60		Bin 5: 0.85–0.87	
	Baseline	Model 3	Baseline	Model 3
	
	Intercept-only	Non-linear growth with random slope	Intercept-only	Linear growth with random slope
	
	B (s.e.)	B (s.e.)	B (s.e.)	B (s.e.)
**Fixed effect**				
Constant	0.86*** (0.01)	0.82*** (0.01)	0.65*** (0.004)	0.61*** (0.01)
Time		0.0015 (0.01)		0.015*** (0.002)
Time^2^		0.006** (0.002)		
**Random effect**				
Intercept variance	0.005	0.010	0.002	0.005
Residuals	0.012	0.010	0.0085	0.0072
Slope variance (time)		0.0003		0.0002
Covariance between intercept and slope		−0.001		−0.0008
Log-likelihood	604.55	664.52	797.17	827.42
LR test (Δ df)		16.56*** (2) vs. model with non-linear growth and fixed slope		9.23** (2) vs. model with linear growth and fixed slope
ICC	0.31		0.22	

***p < 0.001, **p < 0.01.

The results of the analysis of the RT changes in the two ratio bins in Cohort 1 (grades 1–5) are presented in Table 10. The results of the analysis of the pattern of RT changes in the easiest ratio revealed that from grade 1 to grade 5, the RT decreased according to a linear pattern. The model with a random slope fit the data better; thus, there was significant between-individual variability in the rate of change in the RT in the easiest ratio. The analysis also demonstrated that the RT significantly decreased in the hardest ratio bin according to a linear pattern. In general, the patterns of change did not significantly differ between the easiest and hardest ratio bins in Cohort 1 (grades 1–5) (Supplementary Figure 7).

**TABLE 10:** Cohort 1: results of ME growth models for changes in ANS RT (in sec.) for the easiest (0.30–0.60) and hardest (0.85–0.87) ratio bins from grade 1 to grade 5.

Variables	Bin 1: 0.30–0.60		Bin 5: 0.85–0.87	
	Baseline	Model 3	Baseline	Model 3
	
	Intercept-only	Linear growth with random slope	Intercept-only	Linear growth with random slope
	
	B (s.e.)	B (s.e.)	B (s.e.)	B (s.e.)
**Fixed effect**				
Constant	1.21*** (0.01)	1.50*** (0.02)	1.25*** (0.02)	1.51*** (0.03)
Time		−0.14*** (0.01)		−0.12*** (0.01)
Time^2^				
**Random effect**				
Intercept variance	0.027	0.12	0.05	0.19
Residuals	0.12	0.06	0.14	0.08
Slope variance (time)		0.005		0.01
Covariance between intercept and slope		−0.02		−0.04
Log-likelihood	−468.83	−200.31	−605.33	−434.36
LR test (Δ df)		102.08*** (2) vs. model with linear growth and fixed slope		102.94*** (2) vs. model with linear growth and fixed slope
ICC	0.19		0.27	

***p < 0.001.

The results of the analysis of the changes in the RT of the correct answers in the two ratio bins in Cohort 2 (grades 5–9) are presented in Table 11. The results indicated that the RT in the easiest ratio decreased from grade 5 to grade 9 according to a non-linear pattern as follows: from grade 5 to grade 7, the RT significantly decreased, but these changes slowed thereafter. This pattern was also identified in the hardest ratio bin. In general, in Cohort 2, the patterns of changes in the RT did not significantly vary between the easiest and hardest ratio bins (Supplementary Figure 8). However, there was a tendency of increasing differences in RT between the two ratio bins.

**TABLE 11:** Cohort 2: results of ME growth models for changes in ANS RT (in sec.) for the easiest (0.30–0.60) and hardest (0.85–0.87) ratio bins from grade 5 to grade 9.

Variables	Bin 1: 0.30–0.60		Bin 5: 0.85–0.87	
	Baseline	Model 3	Baseline	Model 3
	
	Intercept-only	Non-linear growth with random slope	Intercept-only	Non-linear growth with random slope
	
	B (s.e.)	B (s.e.)	B (s.e.)	B (s.e.)
**Fixed effect**				
Constant	0.87*** (0.01)	1.03*** (0.02)	0.94*** (0.01)	1.07*** (0.02)
Time		−0.15*** (0.01)		−0.15*** (0.02)
Time^2^		0.023*** (0.003)		0.03*** (0.004)
**Random effect**				
Intercept variance	0.01	0.035	0.02	0.05
Residuals	0.036	0.021	0.05	0.03
Slope variance (time)		0.002		0.003
Covariance between intercept and slope		−0.007		−0.009
Log-likelihood	136.51	277.65	−26.88	34.16
LR test (Δ df)		63.31*** (2) vs. model with non-linear growth and fixed slope		37.89*** (2) vs. model with non-linear growth and fixed slope
ICC	0.22		0.30	

***p < 0.001.

### How Do the Changes in ANS Accuracy and RT Relate to Each Other?

Next, we estimated the correlations between individual changes in ANS accuracy and RT. For each individual, the deviations from the average value of the time changes in accuracy and RT were calculated. Positive individual deviation values for accuracy indicated that the individual had a larger growth in ANS accuracy than the sample mean. Positive individual deviation values for ANS RT indicated that the individual had a slower decrease in ANS RT than the sample mean.

In Cohort 1 (grades 1–5), the correlation between the individual deviation in accuracy and RT was negative (*r* = −0.18, *p* < 0.001). This finding indicated that the individuals who demonstrated a faster decrease in RT had a greater increase in accuracy, although the correlation was weak (Supplementary Figure 9).

In Cohort 2 (grades 5–9), there were significant individual differences in the slopes of the variables “time” and time^2^” using the RT as the outcome; thus, two correlation coefficients were estimated. The individual deviations in the slope of the variable “time” in accuracy and RT were positively correlated (*r* = 0.44, *p* < 0.001), whereas the correlation between the individual slope of “time” in accuracy and the slope of “time ^2^” in RT was negative (*r* = −0.34, *p* < 0.001). This finding indicated that the individuals who demonstrated a greater growth in accuracy had a smaller decrease in RT, but they exhibited less deceleration in the RT changes (Supplementary Figure 10).

Notably, the correlation between the changes in accuracy and RT in Cohort 1 (grades 1–5) was weaker than that in Cohort 2 (grades 5–9).

### How Do the Changes in General PS Correlate With the Changes in ANS Accuracy and RT?

To estimate the extent to which the changes in general PS explain the changes in ANS accuracy and RT, we added general PS as a predictor of ANS accuracy and RT. The results are presented in Table 12. The analysis revealed that in Cohort 1, the changes in RT and accuracy were partially explained by the changes in general PS, although the changes in both accuracy and RT remained significant. A faster general PS was positively associated with higher accuracy and smaller RT in the non-symbolic comparison test. In Cohort 2, general PS was not correlated with RT in the ANS test and did not explain the changes in RT but was significantly correlated with accuracy.

**TABLE 12:** Results of ME growth models for changes in ANS accuracy and RT with general PS (in sec.) as a predictor.

Variables	Cohort 1 (grades 1–5)		Cohort 2 (grades 5–9)	
	Accuracy	RT	Accuracy	RT
	
	B (s.e.)	B (s.e.)	B (s.e.)	B (s.e.)
**Fixed effect**				
Constant	0.63*** (0.01)	1.48*** (0.03)	0.68*** (0.01)	1.07*** (0.02)
Time	0.03*** (0.005)	−0.12*** (0.01)	−0.001 (0.005)	−0.14*** (0.02)
Time^2^	−0.004** (0.001)		0.004*** (0.001)	0.02*** (0.003)
General PS (in Z-scores)	−0.007* (0.003)	0.025* (0.01)	−0.02*** (0.003)	−0.02 (0.05)
**Random effect**				
Intercept variance	0.004	0.15	0.005	0.04
Residuals	0.004	0.06	0.003	0.02
Slope variance (time)	0.0002	0.01	0.0002	0.02
Slope variance (time^2^)				0.001
Covariance between intercept and slope (time)	−0.0005	−0.03	−0.0006	−0.02
Covariance between intercept and slope (time^2^)				0.002
Covariance between slope (time) and slope (time^2^)				−0.004
Log-likelihood	1273.99	−242.72	1111.80	234.17
LR test (Δ df)	6.38* (1) (vs. Model 3)	5.31* (1) (vs. Model 3)	45.80*** (1) (vs. Model 3)	0.24 (1) (vs. Model 3a)

***p < 0.001, **p < 0.01, *p < 0.05.

Next, we estimated the developmental changes in general PS in Cohort 1 (Supplementary Table 4) and Cohort 2 (Supplementary Table 5). The analysis revealed that general PS increased from grade 1 to grade 5 according to a non-linear pattern and that there was significant between-individual variability in the rate of change. The results of Cohort 2 revealed that general PS improved from grade 5 to grade 9 according to a linear pattern.

**FIGURE 3:**
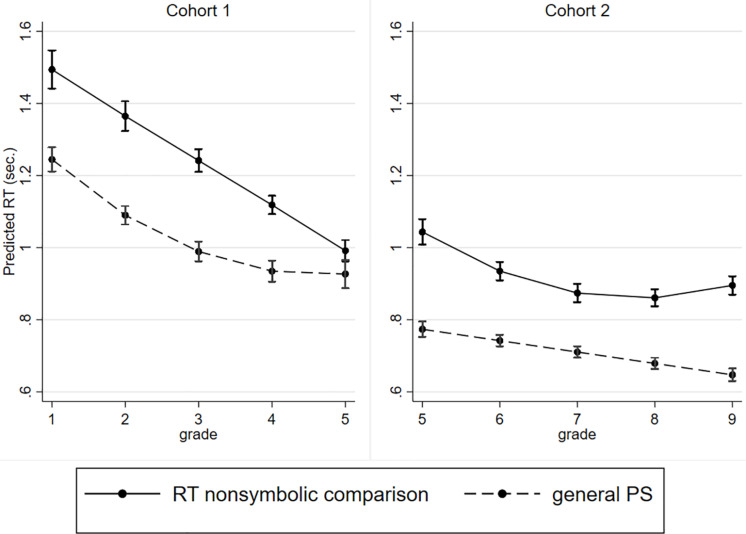
Developmental changes in RT for the ANS test and for the general PS test (in second) with 95% CI.

Next, we compared the patterns of changes in non-symbolic and general PS (Figure 3). The analysis revealed that general PS changed in a non-linear pattern from grade 1 to grade 5, whereas non-symbolic PS changed in a linear pattern. In contrast, in Cohort 2, general PS changed linearly, whereas non-symbolic PS changed non-linearly. Notably, there was a significant difference in RT in the general PS test in grade 5 between Cohort 1 and Cohort 2.

## Discussion

This study aimed to estimate developmental changes in ANS precision from grade 1 to grade 5 and from grade 5 to grade 9 using longitudinal data from two cohorts of Russian children. Previously, investigations of the development of ANS precision were mostly based on evaluations of accuracy (e.g., Tikhomirova et al., 2019) or the Weber fraction, which is an indicator of ANS precision that is highly correlated with accuracy (e.g., Halberda et al., 2012; Inglis and Gilmore, 2014; Tosto et al., 2017). Less often, studies have used measures based on RT to estimate age-related differences in the ANS (Halberda et al., 2012). However, following the findings of previous studies using different measures of ANS precision (e.g., Dietrich et al., 2015, 2016), we assumed that an inspection of the developmental patterns of both accuracy and RT in non-symbolic comparison tests might provide important insight ANS development. Hence, we inspected the developmental patterns of ANS precision using two measures, i.e., the proportion of correct answers and mean RT of correct answers. To account for one of the main features of non-symbolic representations, i.e., ratio dependence, we also estimated the mean RT and accuracy in five ratio bins separately. We aimed to compare the developmental patterns of accuracy and RT between the smallest (easiest) ratio and the largest (hardest) ratio.

The analysis revealed that accuracy decreased as the ratio between the two compared sets increased, and in the largest ratio, accuracy was significantly lower than that in the smallest ratio. Furthermore, the difference in RT between the ratio bins was less impressive than that in accuracy. This finding indicated that the sensitivity to increasing ratios between the compared arrays manifested in decreasing accuracy, but the RT changed to a lesser extent.

The estimation of the developmental changes in accuracy in the two cohorts revealed that accuracy increased from grade 1 to grade 3 and from grade 7 to grade 9 but did not significantly change from grade 3 to grade 7. In both cohorts, the model with the quadratic patterns of changes fit the data better than the model with linear changes. The pattern of quadratic changes in Cohort 1 (grades 1–5) indicated faster growth in ANS accuracy and then slower changes. In Cohort 2 (grades 5–9), the opposite pattern was found as follows: the insignificant growth from grade 5 to grade 7 was replaced by growth in accuracy from grade 7 to grade 9. The analysis also revealed significant inter-individual changes in the rate of change in accuracy in both cohorts. Notably, the obtained quadratic pattern of changes fit better than the linear pattern in a restricted period only. The generalization of these patterns of changes to a wider period should be performed with caution. Quadratic models imply U-shaped trajectories in development, but this trajectory can manifest later in development. In this study, the quadratic pattern revealed that growth slowed (Cohort 1) or accelerated (Cohort 2).

The RT significantly decreased from grade 1 to grade 5 in a linear pattern. In the second cohort (grades 5–9), the changes in RT followed a non-linear pattern as follows: these changes occurred more rapidly from grade 5 to grade 7 and then slowed. Our study confirmed that ANS accuracy increased and RT decreased across development; thus, at the end of secondary school, the pupils demonstrated higher accuracy and shorter RT than the first-graders. These results are consistent with several studies demonstrating that adults have lower RTs and higher accuracy in ANS tests (e.g., Halberda et al., 2012). Furthermore, the period during which accuracy and RT change in one direction (increase in accuracy and decrease in RT) take turns with periods during which changes in RT may continue, while accuracy is stabilized, and vice versa.

The combination of changes in ANS accuracy and RT allows us to identify three stages of developmental changes in ANS precision across 9 years of formal schooling. The first stage (grade 1–grade 3, age from 7 to 9 years) was characterized by faster increases in accuracy and speed of non-symbolic comparison. During the second stage (grade 3–grade 7, age from 9–13), accuracy stabilized, while the speed of non-symbolic comparisons continued to increase. During the third stage (grade 7–grade 9, 13–15 years), ANS accuracy started to increase again, while ANS RT did not significantly change.

These findings indicate that at different developmental stages, changes in the precision of ANS manifest in different ANS measures, which should be considered. It is possible that at the beginning of formal education, changes in the precision of ANS manifest in both accuracy and RT, but later, growing precision mostly manifests in decreased RT but not increased accuracy. At the end of secondary school (grades 7–9, age range 13–15 years), in turn, changes in RT might not reflect changes in ANS precision, whereas growth in accuracy might indicate growth in ANS precision during this stage of development.

Although we did not directly estimate the NRE and its changes, we can compare the developmental trajectories between the easiest and hardest ratio bins. The inspection of the changes in accuracy in the two ratio bins revealed that from grade 1 to grade 5, the changes in the easiest ratio bin were larger than those in the hardest ratio bin, although in both ratio bins, non-linear patterns of changes were identified. In Cohort 2 (grades 5–9), the patterns of the change in accuracy differed between the two ratio bins. The easiest ratio changes followed a non-linear pattern with acceleration of growth, while accuracy in the hardest ratio bin changed linearly. Notably, in both cohorts, the changes in accuracy were more prominent in the easiest ratio. This finding might indicate that the increased accuracy in the non-symbolic comparison test was driven by an increase in accuracy in easier tasks. These results are likely to indicate a slight increase in the NRE as this increase occurs on account of growth in accuracy in trials with the easy ratio.

There are controversial findings regarding the development of the NRE. Some studies have demonstrated that the NRE is reduced across age (Holloway and Ansari, 2009), while other studies have demonstrated increases in the NRE (Lyons et al., 2015). Several studies also found that the NRE or NDE were stable across time (Reynvoet et al., 2009; Defever et al., 2011). The differences in the obtained findings might be related to different formats of magnitude (symbolic or non-symbolic), different types of tasks (priming vs. comparison) or different formats of stimulus presentation (paired vs. intermixed format) in the non-symbolic comparison task. Particularly, it has been demonstrated that the NRE under paired conditions was stronger than that under intermixed conditions (Price et al., 2012). It has also been demonstrated that the distance effect in priming tasks was stable across age (Defever et al., 2011), while the distance effect in comparison tasks decreased (Holloway and Ansari, 2008). In general, it might be concluded that the NRE is sensitive to the format of tasks and cannot be considered a reliable measure of ANS precision and its development.

In addition, the results of the current study indicated that accuracy and RT had different levels of inter-individual variability. The ICC value of accuracy was higher than that of RT in both cohorts (for accuracy, the ICC value was 0.36 and 0.39 in Cohort 1 and Cohort 2, respectively, whereas for RT, the ICC value was 0.26 and 0.31). This finding indicates that individuals exhibited variations in accuracy in the ANS test to a greater extent than they exhibited variations in RT and that RT was a less stable measure of ANS precision than accuracy.

The different roles of accuracy and RT were considered within the diffusion model (Ratcliff, 2002; Park and Starns, 2015; Ratcliff et al., 2016). The diffusion model considers each task a decision process that can be performed based on the noisy accumulation of information. Several components of decision processes were identified, including the drift rate (the rate of the accumulation of information available for use in a decision), boundary settings (boundary of correct or incorrect responses) and non-decision processes. Ratcliff et al. (2015) demonstrated that in numerical tasks, accuracy is largely determined by the drift rate, whereas the RT is determined by boundary settings. It was also shown that the slower RT of children than that of young adults could be explained by wider boundary separation and non-decision processes. For example, the reduction in the RT of older children compared to that of first-graders might be related to a decrease in the amount of time devoted to non-decision processes, such as stimulus encoding and response execution (Ratcliff et al., 2012).

It is possible to assume that the changes in accuracy and RT can be explained by different factors. The faster growth in ANS accuracy at the start of formal schooling might be associated with the acquisition of symbolic math skills and math knowledge, which may facilitate ANS development. Evidence suggests that education has a significant effect on ANS precision and that symbolic representation predicts the precision of non-symbolic representation (e.g., Piazza et al., 2013; Mussolin et al., 2014; Shusterman et al., 2016). In addition, pupils start to receive regular feedback from their teachers and parents during grades 1–2. Previous studies have demonstrated that feedback may improve ANS precision (e.g., DeWind and Brannon, 2012). Thus, children have the opportunity to adjust the system of non-symbolic representation at the start of formal schooling during the acquisition of symbolic math skills, and receiving feedback contributes to improvements in ANS precision.

The improvement in non-symbolic comparison might reflect the progressive automatization of access to non-symbolic representation. Ample evidence highlights the involvement of the IPS in the processing of numerosity in both symbolic and non-symbolic formats (e.g., Hubbard et al., 2008; Holloway and Ansari, 2010). It has been demonstrated that the involvement of the IPS in processing symbolic and non-symbolic numerosity increases across age (Ansari et al., 2005; Ansari and Dhital, 2006; Hubbard et al., 2008), while the activation of frontal areas decreases (e.g., Gullick and Wolford, 2013). Many studies have demonstrated a frontoparietal shift in numerical cognition, which likely reflects less recruitment of frontal areas associated with attention, working memory, and executive functions (Ansari et al., 2005; Rivera et al., 2005). Evidence indicates that slower individuals may require more prefrontal executive control than faster individuals to perform successfully (Rypma et al., 2006). Therefore, an increase in non-symbolic PS might reflect a reduced involvement of frontal areas during non-symbolic comparisons.

The difference in the mechanisms supporting changes in accuracy and RT was demonstrated in several studies of non-numerical processing. In particular, Santee and Egeth (1982) postulated that accuracy and RT reflect different perceptual processes in letter recognition tasks. Accuracy is more sensitive to the early perceptual stage of processing, whereas the RT is more sensitive to later perceptual processing. This difference was also confirmed in studies involving other perceptual and attentional tasks. For example, in somatosensory discrimination tasks, attentional cues have been found to affect accuracy and RT via different cognitive and neural processing methods (van Ede et al., 2012). The cueing effect on accuracy was explained by a preparatory process (increasing activity in the somatosensory cortex) only, whereas the effect of RT was additionally explained by a post-target process. Perri et al. (2014) conducted an EEG study involving an execution go/no-go task and demonstrated that speed and accuracy are processed by two interacting but separate neurocognitive systems. The authors identified groups of individuals according to their tendency to prefer speed or accuracy and considered event-related potential (ERP) components after a stimulus to highlight the different levels of perceptual processing-supported speed or accuracy tendency. It was demonstrated that baseline activity (before the stimulus appearance) in the supplementary motor area differentiates “speedy” and “slow” individuals, whereas activation of the right prefrontal cortex differentiates “accurate” and “inaccurate” groups. The analysis of post-stimulus activity revealed a difference in the P1 ERP component between the faster and slow groups and a difference in the N1 ERP component between the accurate and inaccurate groups. Considering the aforementioned studies, it is possible that differences in developmental changes in accuracy and RT in non-symbolic comparisons to some extent reflect differences in the maturation and development of two distinct neurocognitive systems. This suggestion can be verified in future longitudinal and neurophysiological studies.

In general, our findings confirm the results of previous studies demonstrating that RT-based measures do not reflect ANS precision in the same way as accuracy-based measures (Dietrich et al., 2016). Although the RT decreased over time, the interpretation of a faster RT as an indicator of a more precise ANS needs to be clarified. The present analysis revealed that in Cohort 1, the improvements in accuracy and speed were positively correlated; thus, the pupils who demonstrated higher growth in accuracy also demonstrated a higher rate of change in the RT. In the second cohort, the opposite pattern was revealed. The pupils who had a greater increase in accuracy demonstrated a lower rate of change in the RT. This finding might indicate that although a lower RT corresponded to older participants from a developmental perspective, it does not always reflect increased accuracy in non-symbolic representation.

This study also revealed that general PS and speed in non-symbolic comparison tasks increased across age. The improvement in both general and non-symbolic PS might be explained by the processes of neuronal axon myelination and synaptic pruning (the process of synapse elimination) (Travis, 1998; Chechik et al., 1999). The myelination of neurons results in more rapid neural computation through faster propagation of action potentials (Mabbott et al., 2006; Fields, 2008; Chevalier et al., 2015). It has also been shown that individual differences in general PS might be associated with regional connectivity, implying a central role of axonal structures in inter-individual activation differences (Rypma et al., 2006). Synaptic pruning leads to a reduction in unused pathways and the strengthening of used pathways (e.g., Chechik et al., 1998). It has been postulated that the process of pruning is driven by individual experience and allows an individual to respond faster to the unique environment in which s/he grows (e.g., Tierney and Nelson, 2009).

However, general PS and non-symbolic PS develop at different rates in different patterns. In Cohort 1, linear changes in non-symbolic RT and non-linear changes in general PS were identified. In Cohort 2, the opposite patterns were observed as follows: general PS developed linearly, while the RT in the non-symbolic comparison changed non-linearly. Moreover, in both cohorts, the changes in general PS did not eliminate the time changes in non-symbolic comparison RT. In addition, general PS was not associated with RT in the non-symbolic comparison in the pupils in grade 5 to grade 9. These findings might confirm the local trend hypothesis of PS development.

It is possible that the development of general PS forms the basis for the development of non-symbolic PS. For example, it has been shown that training in PS led to improvements in other cognitive functions (Takeuchi and Kawashima, 2012). The patterns of change in non-symbolic PS repeated the developmental patterns of general PS at a previous age. However, the opposite relationships might also exist, i.e., the development of general PS might combine the development of specific processes. To verify this suggestion, it is necessary to include more time points in longitudinal data and additional different tasks for the estimation of PS in different processes.

Notably, in this study, general PS was more correlated with accuracy than RT in the non-symbolic comparison test. On the one hand, these results might reflect the close relationships between general PS and other cognitive constructs measured by accuracy. For example, many studies have demonstrated that general PS is associated with intelligence and working memory (Fry and Hale, 1996; Sheppard and Vernon, 2008). Moreover, it has been shown that general PS is substantially correlated with untimed tests (Wilhelm and Schulze, 2002). It is possible that the association between accuracy in the non-symbolic comparison test and general PS is not explained by time restriction during the execution of a non-symbolic comparison test.

On the other hand, the association between accuracy in a non-symbolic comparison test and general PS can be explained by the specificity of the general PS test, which was considered in the current study. In the test used in the present study, the children were asked to press a key corresponding to a digit (1, 2, 3, or 4) appearing on the screen as fast and accurately as possible. The mean RT of the correct answers was used as an indicator of general PS. Therefore, symbolic math skills were utilized to some extent to execute this test. The link between the results of the RT test and the accuracy of the non-symbolic comparison test might be partially explained by their association with symbolic math skills.

The current study had some limitations regarding the test used for the estimation of ANS. Some authors suggest that in tasks involving non-symbolic comparison, individuals are affected by the visual properties of the arrays. Arrays of objects can be compared based on comparisons of visual properties, such as cumulative area or convex hull (Gebuis and Reynvoet, 2012; Gebuis et al., 2016). To confirm the effect of visual properties on accuracy in comparisons of two sets of dots, researchers have manipulated different visual properties and identified two types of trials. The first type was congruent trials in which the visual properties were positively correlated with the magnitude. The second type was incongruent trials in which the magnitude was negatively correlated with the visual properties (e.g., Gebuis and Reynvoet, 2012; Clayton et al., 2015; Gilmore et al., 2016). It was demonstrated that accuracy in such comparisons was higher and the RT was faster in congruent trials than incongruent trials (congruency effect) (e.g., Gebuis and Reynvoet, 2012; Szucs et al., 2013). The congruency effect was used to confirm that numerosity judgments are based on the estimation of the visual properties of stimuli (Gebuis and Reynvoet, 2012).

In the current version of the ANS test, all trials in the test were congruent, and the array that contained more dots had a larger cumulative area. Hence, this version of the ANS test can measure accuracy in both non-symbolic representation and estimation of visual cues. It has been shown that activation of brain areas involved in numerical processing does not significantly differ between congruent and incongruent trials (Wilkey et al., 2017). This finding might indicate that even in congruent trials, individual can estimate numerosity in parallel with visual cues. Moreover, we used a “blue-yellow dots” test with an intermixed format, and it has been demonstrated that the reliability of this test in the intermixed format is higher than that in the paired or sequential formats (Price et al., 2012). Based on previous findings, we propose that the obtained results reflect the developmental trends in non-symbolic comparisons to a large extent.

We also used the same version of the test each year. This approach has some advantages, such as the ability to directly compare accuracy and RT across years. The period between testing was relatively long (nearly 1 year), and feedback was not provided; thus, we can avoid the effect of memory or training on the results of the test.

## Conclusion

This study is the first to estimate the longitudinal development of ANS precision based on an inspection of changes in both accuracy and RT. Our findings revealed that the developmental patterns of changes in ANS accuracy and RT were not synchronous, but an inspection of both measures might provide new insight into ANS development.

In general, three stages of ANS development were identified. During stage 1 (grade 1–grade 3, age 7–9 years), development was characterized by faster growth in accuracy and non-symbolic PS. Stage 2 (grade 3–grade 7, age 9–13 years) was characterized by stability in accuracy and continuing increases in non-symbolic PS. During stage 3 (grade 7–grade 9, age 13–15), the opposite trend was revealed, i.e., accuracy started to increase, while PS stabilized. A speed-accuracy trade-off was identified at all time points. In general, the results of this study suggest that for a more informative investigation of ANS development, an inspection of both accuracy and RT is needed.

## Data Availability Statement

The raw data supporting the conclusion of this article will be made available by the authors, without undue reservation.

## Ethics Statement

The studies involving human participants were reviewed and approved by Ethics Committee of the Psychological Institute of the Russian Academy of Education. Written informed consent to participate in this study was provided by the participants’ legal guardian/next of kin.

## Author Contributions

SM directs and received funding for the “Cross-cultural Longitudinal Analysis of Student Success” (CLASS) project. SM and TT conceived and designed the present study. YK and TT conducted the analyses and interpreted the results under the supervision of SM. YK drafted the manuscript. All authors discussed the results and implications and provided comments regarding the manuscript at all stages. All authors approved the final version of the manuscript for submission.

## Conflict of Interest

The authors declare that the research was conducted in the absence of any commercial or financial relationships that could be construed as a potential conflict of interest.
