# Supplementary material for: Developmental Changes in ANS Precision Across Grades 1–9: Different Patterns of Accuracy and Reaction Time
Source: Front Psychol. 2021 Mar 24;12:589305. doi: 10.3389/fpsyg.2021.589305 (PMC8024480; doi:10.3389/fpsyg.2021.589305)
Supplement: Supplementary file 1 [file Data_Sheet_1.pdf]

Supplementary materials

Table 1

Predicted average accuracy with 95% CI for Cohort 1 (from quadratic models)

**Grades**	**Accuracy**		
	Overall	Ratio bin 0.30 - 060	Ratio bin 0.85 – 0.87
Grade 1	0.62 [0.61; 0.63]	0.74 [0.72; 0.76]	0.58 [0.57; 0.60]
Grade 2	0.65 [0.64; 0.66]	0.79 [0.78; 0.81]	0.60 [0.59; 0.61]
Grade 3	0.68 [0.67; 0.69]	0.83 [0.81; 0.84]	0.62 [0.61; 0.63]
Grade 4	0.69 [0.68; 0.70]	0.85 [0.83; 0.86]	0.63 [0.62; 0.64]
Grade 5	0.69 [0.68; 0.70]	0.85 [0.84; 0.87]	0.63 [0.62; 0.64]

Table 2

Predicted average accuracy and RT for correct answers with 95% CI for Cohort 2 (from quadratic models)

Grades	Accuracy (% of correct answers)		RT for correct answers (in sec.)		
	Overall	Ratio bin 0.30 - 060	Overall	Ratio bin 0.30 - 060	Ratio bin 0.85 – 0.87
Grade 5	0.68 [0.66; 0.69]	0.82 [0.80; 0.84]	1.05 [1.01; 1.08]	1.03 [1.00; 1.07]	1.07 [1.03; 1.11]
Grade 6	0.68 [0.67; 0.69]	0.83 [0.82; 0.85]	0.94 [0.91; 0.96]	0.91 [0.89; 0.93]	0.95 [0.92; 0.98]
Grade 7	0.70 [0.69; 0.71]	0.85 [0.84; 0.87]	0.87 [0.85; 0.90]	0.83 [0.81; 0.85]	0.88 [0.85; 0.91]
Grade 8	0.72 [0.71; 0.73]	0.88 [0.87; 0.89]	0.86 [0.84; 0.88]	0.80 [0.78; 0.82]	0.88 [0.85; 0.90]
Grade 9	0.75 [0.74; 0.76]	0.92 [0.91; 0.94]	0.90 [0.87; 0.92]	0.81 [0.79; 0.84]	0.93 [0.90; 0.96]

Table 3

Descriptive statistics for general PS

**Cohort**	**Grade**	**Reaction time (sec.)**		
		Mean	SD	95% CI
First	Grade 1	1.25	0.29	1.21; 1.29
	Grade 2	1.07	0.24	1.04; 1.10
	Grade 3	1.02	0.31	0.98; 1.06
	Grade 4	0.92	0.27	0.89; 0.95
	Grade5	0.93	0.30	0.89; 0.97
Second	Grade 5	0.78	0.18	0.76; 0.81
	Grade 6	0.74	0.17	0.72; 0.76
	Grade 7	0.70	0.15	0.68; 0.72
	Grade 8	0.69	0.14	0.67; 0.70
	Grade 9	0.65	0.13	0.63; 0.67

Table 4

Cohort 1: Results of ME growth models for changes in general PS (in sec.) from grade 1 to grade 5

**Variables**	**Baseline**	**Model 1**	**Model 2**	**Model 3**
	Intercept-only	Linear growth	Nonlinear growth	Model with random slope
	B (s.e.)	B (s.e.)	B (s.e.)	B (s.e.)
Fixed effect				
Constant	1.03*** (0.01)	1.19*** (0.02)	1.24*** (0.02)	1.24*** (0.02)
Time		-0.08*** (0.005)	-0.17*** (0.02)	-0.17*** (0.02)
Time ^{2}			0.02*** (0.004)	0.02*** (0.004)
Random effect				
Intercept variance	0.03	0.03	0.03	0.04
Residuals	0.07	0.05	0.05	0.04
Slope variance (time)				0.005
Covariance between intercept and slope				-0.006
Log-likelihood	-200.255	-91.19	-73.12	-41.26
LR test (Δ df)		218.13*** (1)	36.14*** (1)	63.73*** (2)
ICC	.30			

***p<.001

Table 5

Cohort 2: Results of ME growth models for changes in general PS (in sec.) from grade 5 to grade 9

**Variables**	**Baseline**	**Model 1**	**Model 2**	**Model 3**
	Intercept-only	Linear growth	Nonlinear growth	Model with random slope
	B (s.e.)	B (s.e.)	B (s.e.)	B (s.e.)
Fixed effect				
Constant	0.71*** (0.01)	0.77*** (0.01)	0.78*** (0.01)	0.77*** (0.01)
Time		-0.03*** (0.002)	-0.05*** (0.01)	-0.03*** (0.002)
Time ^{2}			0.004 (0.002)	
Random effect				
Intercept variance	0.01	0.01	0.01	0.01
Residuals	0.02	0.014	0.014	0.013
Slope variance (time)				0.0005
Covariance between intercept and slope				-0.002
Log-likelihood	492.17	556.71	558.06	562.69
LR test (Δ df)		129.08*** (1)	2.70 (1)	11.96** (2)
ICC	.36			

***p<.001, **p < .01

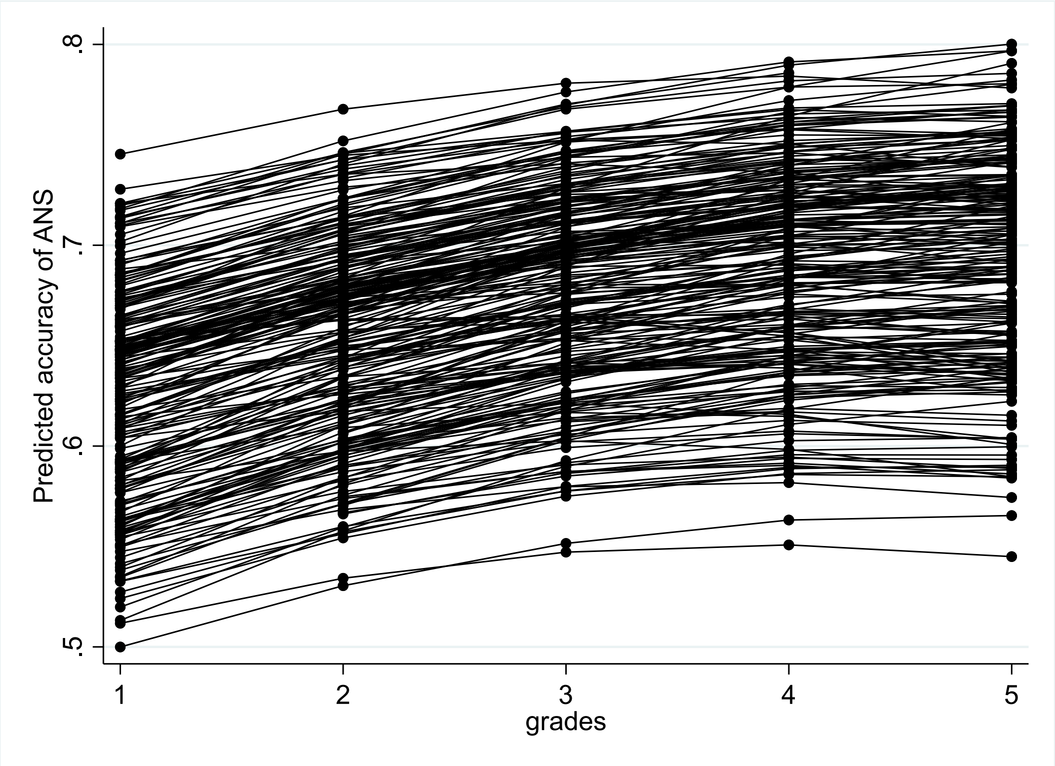

Figure 1. Individual trajectories of the development of ANS accuracy from grade 1 to grade 5 (Cohort 1)

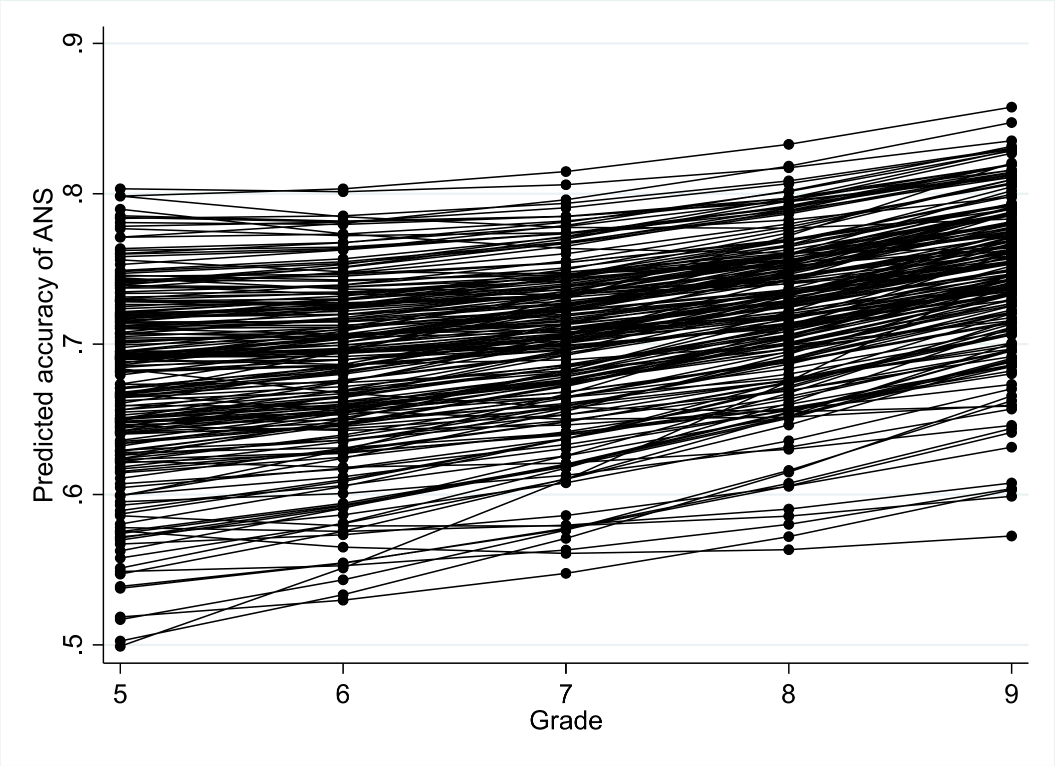

Figure 2. Individual trajectories of the development of ANS accuracy from grade 5 to grade 9

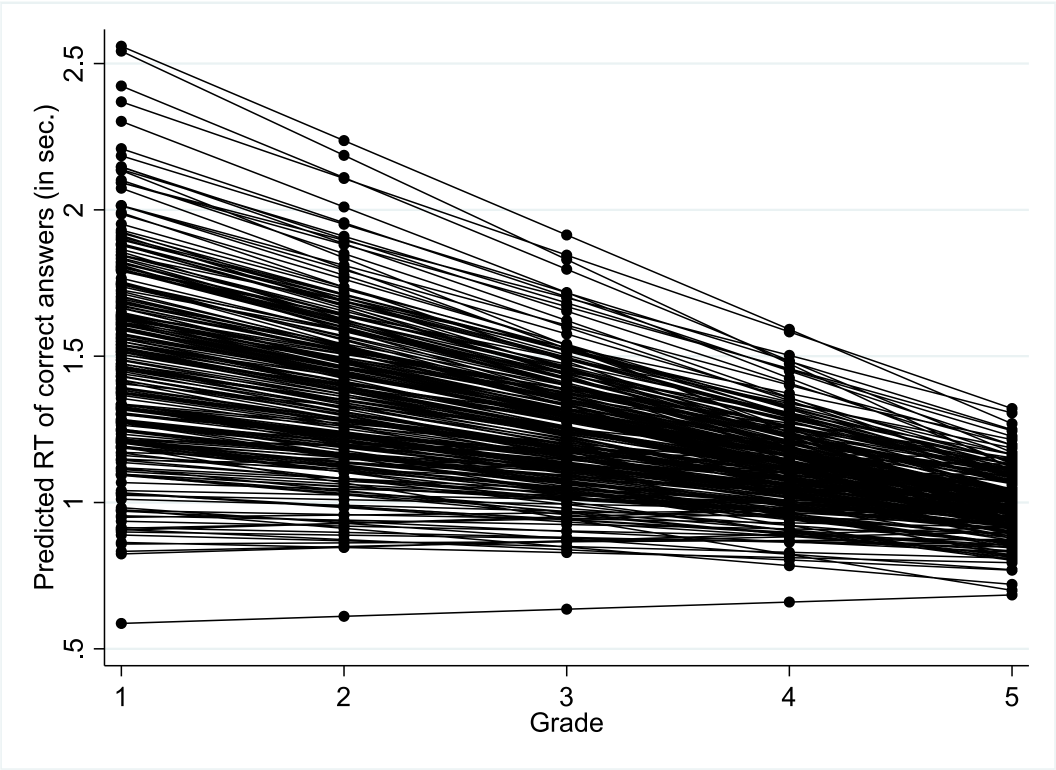

Figure 3. Individual predicted trajectories of changes in ANS RT from grade 1 to grade 5

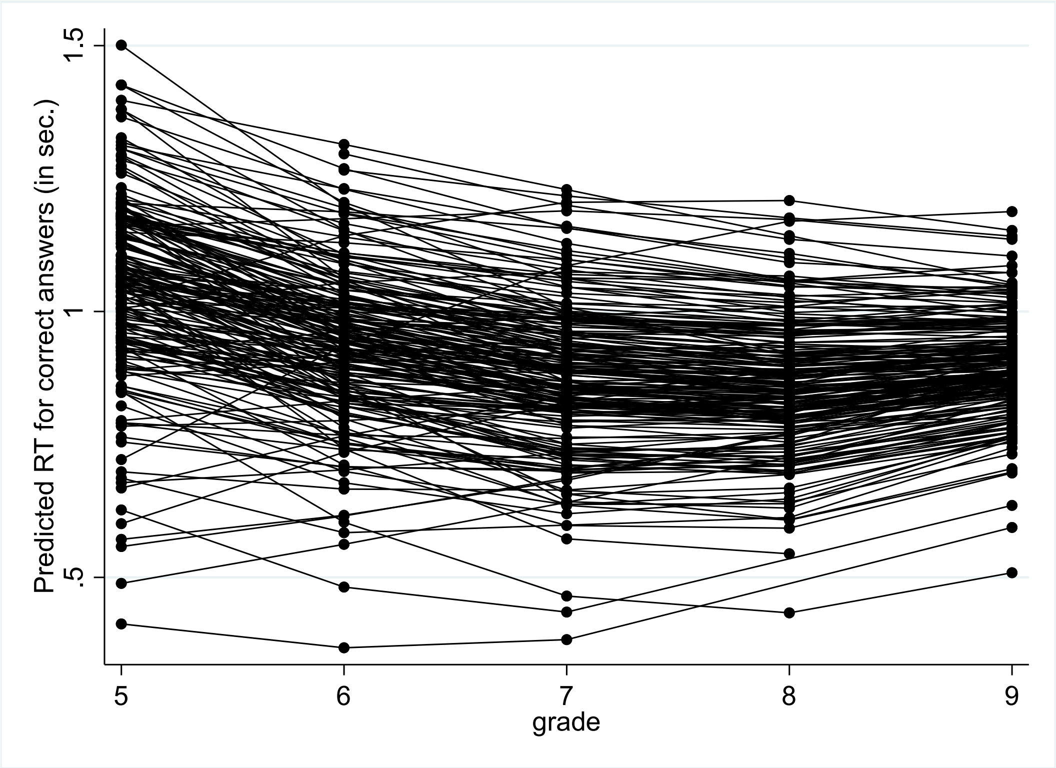

Figure 4. Individual predicted trajectories of changes in ANS RT from grade 5 to grade 9

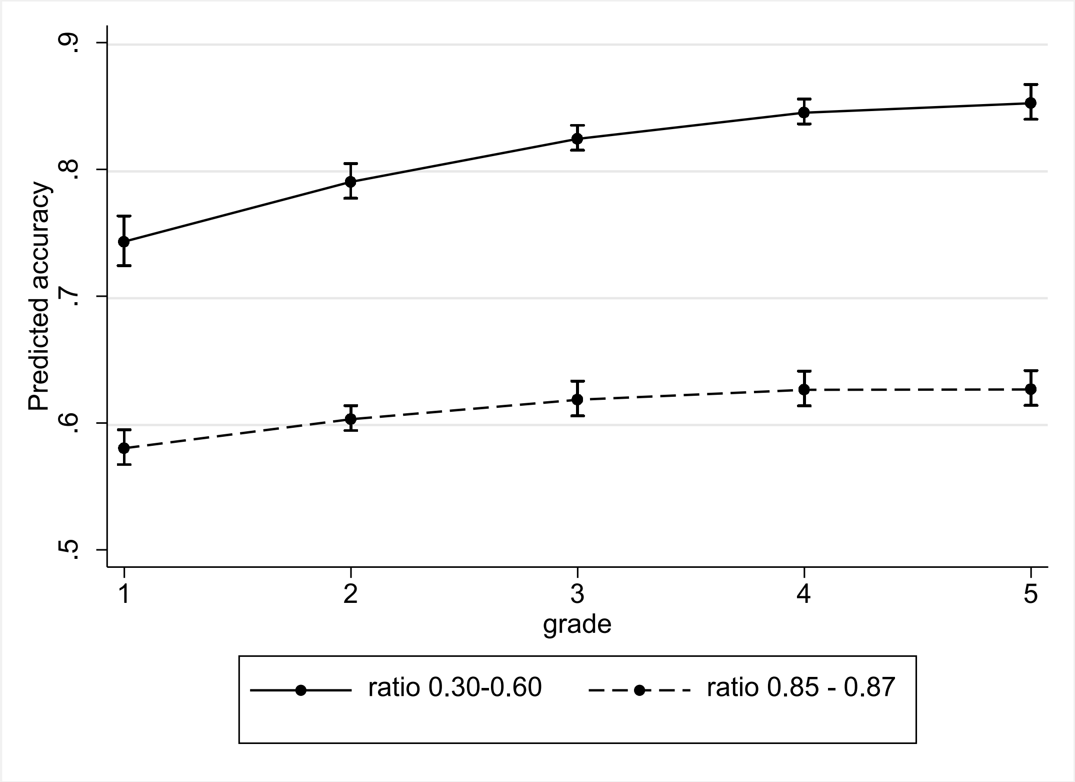

Figure 5. The average growth in ANS accuracy for the easiest and hardest ratio bins with 95% CI for Cohort 1 (grades 1–5)

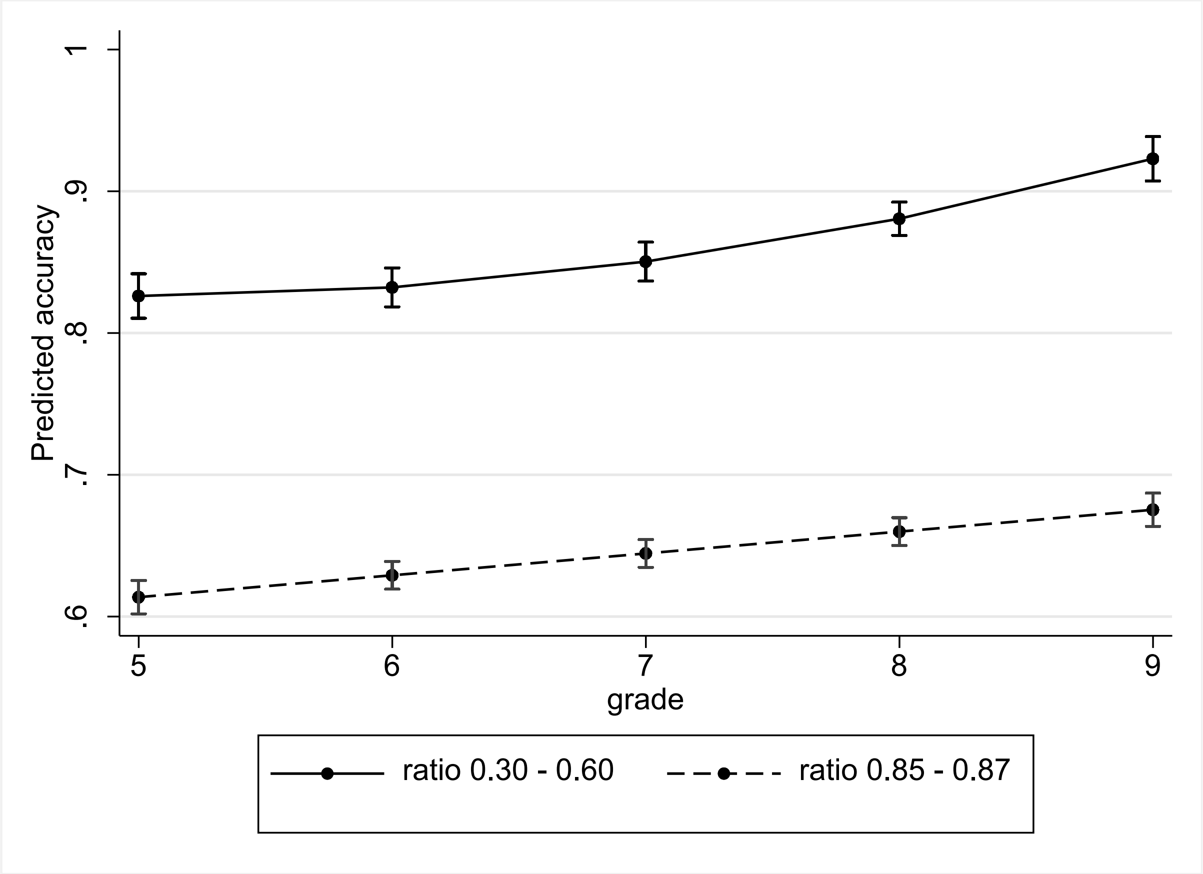

Figure 6. The average growth in ANS accuracy with 95% CI for the easiest and hardest ratio bins for Cohort 2 (grades 5–9)

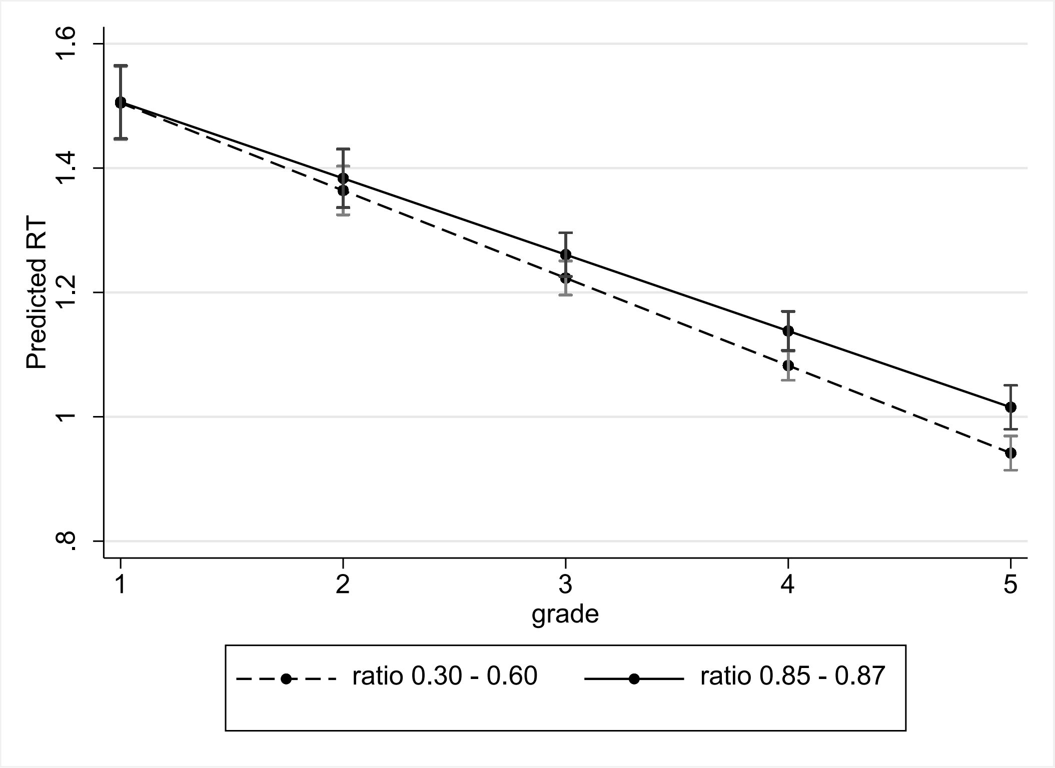

Figure 7. Predicted changes in ANS RT with 95% CI for two ratio bins for Cohort 1 (grades 1–5)

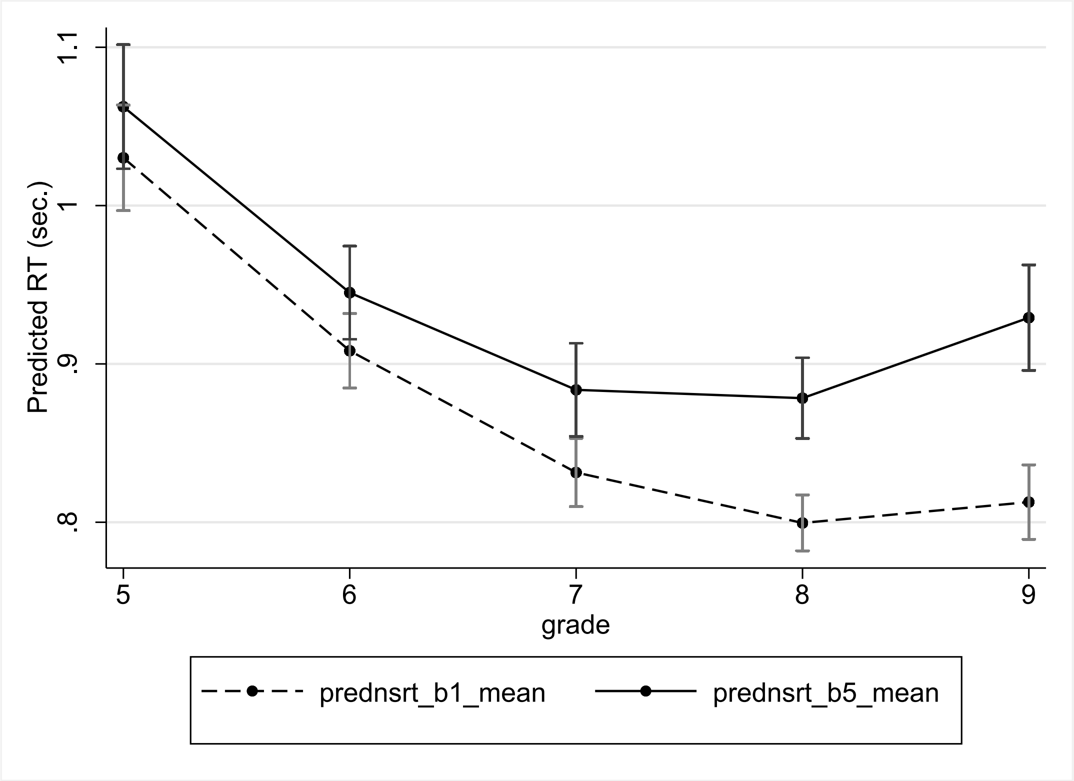

Figure 8. Predicted changes in ANS RT with 95% CI for two ratio bins for Cohort 2 (grades 5–9)

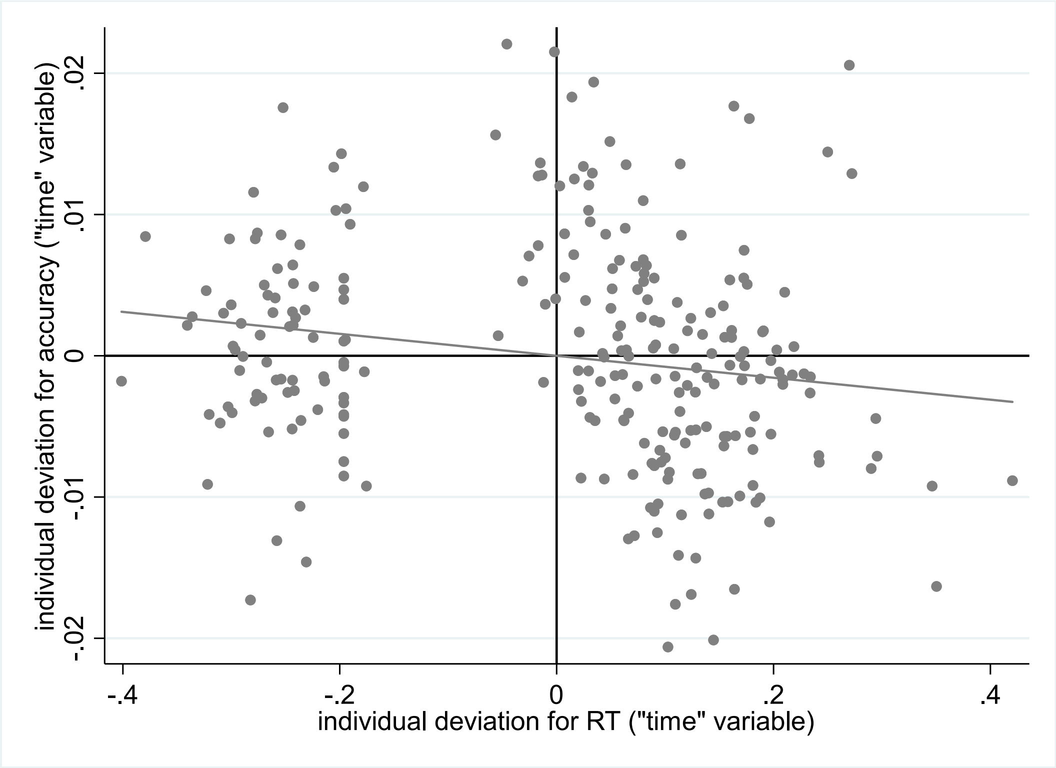

Figure 9. The correlation between individual deviation from the average value of time changes in accuracy and RT for Cohort 1 (grades 1–5)

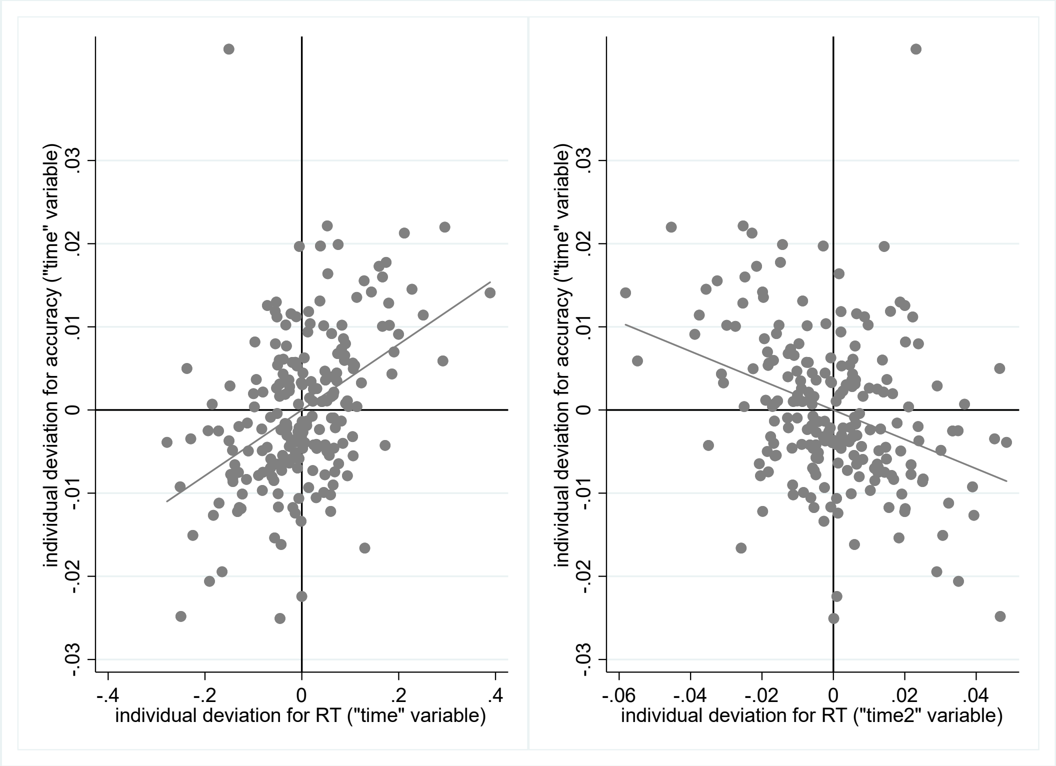

Figure 10. The correlation between individual deviation from the average value of time changes in accuracy and RT for Cohort 2 (grades 5–9)
